# Integrated forecasting and deep reinforcement learning for price-based self-scheduling of PV-BESS: Utility-scale evidence in Chile

**DOI:** 10.1371/journal.pone.0336753

**Published:** 2026-01-09

**Authors:** Juan Pérez, Gustavo Lobos, Milena Bonacic

**Affiliations:** Facultad de Ingeniería y Ciencias Aplicadas, Universidad de Los Andes, Santiago, Chile; Aalto University, FINLAND

## Abstract

Deep Reinforcement Learning (DRL) shows good performance for optimizing battery energy storage systems (BESS) coordinated operations with photovoltaic plants (PV), yet most studies rely on simulations. Bridging the gap to practical application requires validation using real-world operational data. This paper provides such empirical evidence by developing and rigorously evaluating an integrated forecast-and-control framework on three distinct utility-scale PV-BESS assets in Chile. The framework couples a Sequence-to-Sequence (Seq2Seq) LSTM for point forecaster embedded in a probabilistic scenario-generation pipeline of PV generation with nodal prices with DRL agents (Proximal Policy Optimization - PPO and Soft Actor-Critic - SAC) trained on 1,000 generated scenarios per site. Using two years (2022–2023) of operational plant data, meteorology, and market prices, we benchmark DRL policies against theoretical limits (Oracle), a deterministic predict-then-optimize baseline, a scenario-based model predictive control (MPC), and a random Dummy policy over 14-day horizons using a 900/100 train–test split. The Seq2Seq forecaster improves accuracy (e.g., 34.5% reduction in RMSE for prices vs. SARIMAX). We find that the DRL agents consistently outperform the predict–then–optimize baseline, achieving mean 14-day profits near USD 55k, and exhibiting robust, adaptive contracyclical behavior without excessive cycling. Our study provides a reproducible blueprint and empirical validation for data-driven BESS control, demonstrating its practical viability and economic benefits in real-world operating conditions.

## 1 Introduction

The use of renewable energy (such as solar and wind power) is critical to mitigate climate change [1]. The intermittency of these sources interferes with the reliability and stability of the grid, and energy storage systems constitute a key enabling technology to allow a better use of such sources [2]. BESS allows PV plants to store surplus energy and inject it into the grid strategically, transforming them into reliable assets. This capability is especially crucial in markets like Chile, which has good solar resources and faces significant operational and economic challenges [3].

We address a *plant-level, price-based self-scheduling* problem for a PV plant with co-located battery storage. Nodal prices are treated as exogenous signals, and we do not model market clearing, bidding strategies, network constraints, or system-level unit commitment, and economic dispatch. This scope aligns with our real-world use case, where the assets are price-taking and operate under the Chilean *Pequeños Medios de Generación Distribuida* (PMGD) regime (see the regulatory note in Sect 3.3.1), in which plants with certain technical operations limits can operate as we describe in this research.

Deep reinforcement learning (DRL) shows significant promise in optimizing the sequential decision making required to operate PV-BESS plants [4,5], yet a gap persists between simulation-based studies and practical industrial-scale deployment. Most DRL research for energy systems remains confined to simulated environments or small-scale pilots, often failing to capture the complexities and uncertainties encountered in commercial operations, particularly the volatile prices in real-world markets such as the Chilean electricity market [6,7]. This market exemplifies the challenges that often face midday price collapses and curtailment of renewable energy despite a high solar potential [3]. To address this gap, empirical validation is required, and the use of operational data from real PV–BESS plants is essential.

We fill this gap by developing, testing, and benchmarking a data-driven framework for BESS control using real-world data from three photovoltaic plants in Chile. Our methodology couples probabilistic forecasting using sequence-to-sequence LSTMs (Seq2Seq) with DRL-based control agents (PPO and SAC) to maximize revenue under real market conditions. We structure this research around this integration of Seq2Seq within a DRL solution scheme.

This study makes the following key contributions:

- *An integrated forecast and control framework:* We implement a system that integrates a Sequence-to-Sequence (Seq2Seq) LSTM model to produce probabilistic price and generation scenarios with DRL agents (PPO and SAC) for control. We trained these models on a large ensemble of scenarios to ensure robustness against uncertainty.
- *Validation in three PV-BESS plants:* We validate our framework using two years of operational data (2022 and 2023) from three PV plants in Chile. We used nodal prices from the National Electric Coordinator (CEN) and public weather data from NASA POWER, and show the effectiveness of the approach under real economic conditions.
- *Comparison with industry practices:* We evaluated the performance of our DRL agents with respect to the following baselines: a theoretical *Oracle* (with a perfect foresight, that we use just to show the upper bound, despite unreachable it is also useful to discard possible data leakages), a *predict-then-optimize* strategy that emulates current engineering practices, and a *dummy* emulating random decisions (just to show the lower bound). Our results, presented in Sect 4, show that DRL agents consistently outperform the predict-then-optimize and MPC benchmarks, demonstrating economic value.

Finally, this work provides a reproducible framework that uses artificial intelligence to enhance the operational efficiency and economic viability of grid-scale PV-BESS plants. We focus on short-term, data-driven decision support, offering a practical solution for plant operators facing conditions such as the ones described in this research.

## 2 Background and literature review

The growing penetration of renewable energy sources is fundamental for global decarbonization efforts [1]. Among these, PV systems are one of the most important, but their intermittency is a problem for the grid stability. The integration of BESS and PV is a key solution that transforms intermittent PV plants into dispatchable energy assets [2]. In this context, the potential of PV-BESS plants is even more interesting when operators can use and optimize Energy Management Systems (EMS) capable of managing the energy deliveries to the grid depending on the market conditions (price). The effective operation of these systems should focus on the following two main aspects: (i) *accurate short-term forecasting* of photovoltaic generation and nodal prices, and (ii) *optimal real-time control* of BESS charge-discharge cycles. Although our work focuses on improving the operation to increase profits, BESS control is also critical to address technical grid stability problems, such as mitigating overvoltage through cooperative reinforcement learning [8]. Taking these aspects into account, we structure our literature review accordingly.

### 2.1 Forecasting of photovoltaic generation and prices

Accurate forecasting is the foundation for effective planning and operation. Methodologies range from physical models, which require extensive site-specific calibration, to data-driven statistical and machine learning (ML) approaches [9]. Classical ML models such as linear regression, random forests [10,11], and support vector machines [12,13] have been widely applied to learn the mappings from historical meteorological data.

More recently, Deep Learning (DL) models; especially recurrent architectures such as long-short-term memory (LSTM) networks have shown superior performance in capturing complex nonlinear temporal dependencies in energy time-series data [14,15]. This advantage is particularly pronounced when large datasets are available. A recent study confirmed this trend, where a hybrid CNN-LSTM model reduced the Mean Absolute Error (MAE) by 18% compared to conventional ML methods in a utility plant [16]. Recent works focused on forecasting to improve economic outcomes; for example, [17] trained a forecasting model with a loss function directly oriented toward arbitrage profit produces better results than simply minimizing forecast error, reinforcing the tight coupling needed between prediction and decision making.

### 2.2 Optimal control of PV-BESS plants

Once the forecast is done, the next step is to schedule BESS operations, considering that the generation and price are not deterministic, this scheduling cannot be optimal, but the objective is to reach good solutions. The use of Reinforcement Learning for real-time arbitrage was formulated by [18], setting the stage for more advanced data-driven approaches.

#### 2.2.1 Traditional approaches versus AI-based control strategies.

Let us consider as conventional control strategies, the ones that use mathematical optimization. Stochastic programming, for example, provides a framework for optimizing decisions under uncertainty by modeling price scenarios; this approach has been shown to be better than its deterministic benchmarks [19]. Although it serves as the theoretical foundation for the *predict-then-optimize* schemes used as benchmarks in many studies, including ours, its computational requirements limit its application for high-use, short-term and decentralized scheduling. Another common approach is Model Predictive Control (MPC), which uses system models and forecasts to compute an optimal control sequence; this approach effectively handles physical constraints [20]. However, the performance of these methods depends on the accuracy of their forecasts and can be computationally difficult for real-time applications. This is particularly true for stochastic optimization approaches, which can become intractable when dealing with a large number of scenarios required for short-term operational decisions.

Approaches using Artificial Intelligence (AI) can overcome these challenges; in particular, Reinforcement Learning (RL) and Deep Reinforcement Learning (DRL) have been explored. RL, for example, has been proposed as an effective method for the complex day-ahead scheduling of generation with stochastic photovoltaic sources [21]. On the other hand, DRL agents can learn optimal control policies directly from the interaction with an environment, making them robust to uncertainties without the need for an explicit system model [4,22]. This is particularly valuable in real-world markets where DRL-based policies outperform fixed rules [23]. The DRL inherent policies; in particular Advantage Actor Critic (A2C) and Proximal Policy Optimization (PPO) algorithms have been proven to be effective for the continuous action spaces found in energy management problems [24,25]. Recent studies actively compare these state-of-the-art algorithms for energy arbitrage tasks. For example, [26] compared PPO and SAC to schedule a community battery system, finding that the SAC algorithm achieved the best performance. Similarly, other works have used SAC in risk-sensitive frameworks to optimize a trade-off between profit and risk [27].

Several frameworks demonstrate that DRL performance improves significantly when forecasting modules are integrated directly into the agent [28–30]. This integrated approach informs decisions and also improves the agent’s robustness with respect to forecasting errors compared to traditional Model Predictive Control (MPC) [31]. For example, recent work [32] explores integrated scheduling approaches using DRL for PV-BESS, showing promising results based on simulation, but with limited operational contexts. However, validating these integrated AI frameworks using extensive operational data from *multiple, commercial-scale* plants remains a critical gap for practical applications. The successful application of DRL extends beyond photovoltaics, having been validated to coordinate wind and storage plants in the energy and ancillary service markets [33], underscoring the importance of using real market data due to price and weather volatility, which affects profitability [34].

#### 2.2.2 The role of battery degradation.

Some authors have pointed out the relevance of battery degradation in PV-BESS optimization; in fact, they have stated that scheduling decisions that ignore battery aging can lead to suboptimal long-term performance. For example, [35] showed that profits can decrease significantly when degradation is ignored. Some advanced DRL frameworks explicitly incorporate degradation models into the reward function to find a balance between profit and long-term battery health [29,36]. It is relevant to disclose that our work does not explicitly model degradation; instead, we focus on short-term operational decision making, and we recognize its relevance when evaluating long term profitability. Nevertheless, the proposed framework can be extended in future research to incorporate such long-term considerations.

### 2.3 Research gap and contribution of this study

Despite significant progress, a review of the literature reveals several critical gaps that this study aims to address. Our main contributions are presented below, framed within these identified gaps.

To situate our contribution within recent work on storage-backed renewable assets, Table 1 summarizes representative studies that combine optimization, reinforcement learning, and forecasting. The comparison highlights the typical system scale, whether real operational data are used, and how uncertainty is represented, which helps to clarify the specific gap addressed by our PV–BESS case with integrated Seq2Seq forecasting and DRL control across three utility-scale plants.

- *Extensive empirical validation using utility-scale operational data:* Addressing the gap between simulation and practice, this work targets a context in which a substantial portion of existing research is validated using simulated data or in small-scale, non-commercial systems [38,39]. Furthermore, while some works focus on different applications such as community-level optimization with high penetration of EV [40], the specific challenges of grid-connected utility-scale PV-BESS plants operating in volatile spot price markets, such as the one in Chile [41], remain underexplored. Our work directly addresses this by validating our methodology in three distinct commercial plants in Chile, using real operational data, public climate data, and authentic marginal price signals from the national electricity market.
- *Development and validation of an integrated forecast-control framework:* While many studies decouple forecasting and control [35], our work presents and *empirically validates* a tightly coupled system combining a Seq2Seq LSTM forecaster with DRL agents (PPO, SAC). Training on a large ensemble of generated scenarios enhances the policy’s robustness against uncertainty, which we demonstrate through rigorous testing.
- *Benchmarking against baselines:* To assess the practical value of the DRL approach, we establish a comparative framework. We benchmark the performance of DRL agents against multiple strategies, including theoretical limits (*Oracle*) and, crucially, a *predict-then-optimize* scheme emulating current industry practice. This comparison provides clear quantitative evidence of the superior economic performance and operational robustness achieved by adaptive DRL policies under realistic conditions.

**Table 1:** Representative recent studies on control of storage-backed renewable assets and relation to this work.

Reference	System and method	Data/evaluation	Limitations relative to this work
[18]	Single BESS performing price arbitrage using reinforcement learning in wholesale markets.	Historical price series; simulated battery model.	No PV coupling; no explicit probabilistic forecasting; validation based on a single storage asset and not on multiple utility-scale PV–BESS plants.
[29]	Grid-level BESS arbitrage with DRL and an explicit lithium-ion degradation model.	Price data and detailed battery model; evaluation in simulation.	Focus on degradation-aware arbitrage for a standalone BESS; does not integrate PV generation, nor a dedicated probabilistic forecaster, nor multi-plant operational data.
[37]	Deep RL for online scheduling of a PV system with co-located BESS.	Case studies based on historical PV and load profiles.	Single-site setting; forecasting and control are not tightly coupled through a shared scenario ensemble; no comparison against scenario-based optimization baselines.
[38]	RL-based energy-storage management in grid-connected microgrids.	Simulated microgrid with storage; focus on offline vs. online implementation.	Microgrid context with generic storage; no utility-scale PV plants, no explicit market price volatility, and no integrated probabilistic forecasting module.
[26]	Community battery scheduling under uncertain load, PV, and prices using PPO and SAC.	Community-scale case study; synthetic or historical time series.	Community BESS without individual utility-scale PV plants; forecasting is not central and there is no validation across multiple commercial assets.
[33]	DRL for coordination of wind generation and storage in energy and ancillary service markets.	Wholesale market case with wind and storage; evaluation on historical series.	Wind + storage portfolio; different asset type and market participation model; does not address plant-level price-based self-scheduling under regimes such as PMGD.
[32]	Integrated scheduling and optimization of PV–storage systems using DRL.	Simulation-based evaluation with synthetic or processed data.	Demonstrates the value of integrating forecasting and DRL, but in a simulated setting with limited operational context and without multi-plant utility-scale validation.

This paper bridges these gaps by developing and implementing the integrated prediction+control framework described above; we use Proximal Policy Optimization (PPO) and Soft Actor-Critic (SAC) as the DRL control agents. In doing so, we provide a robust and reproducible approach for the management of PV-BESS plants.

## 3 Materials and methods

**Fig 1:**
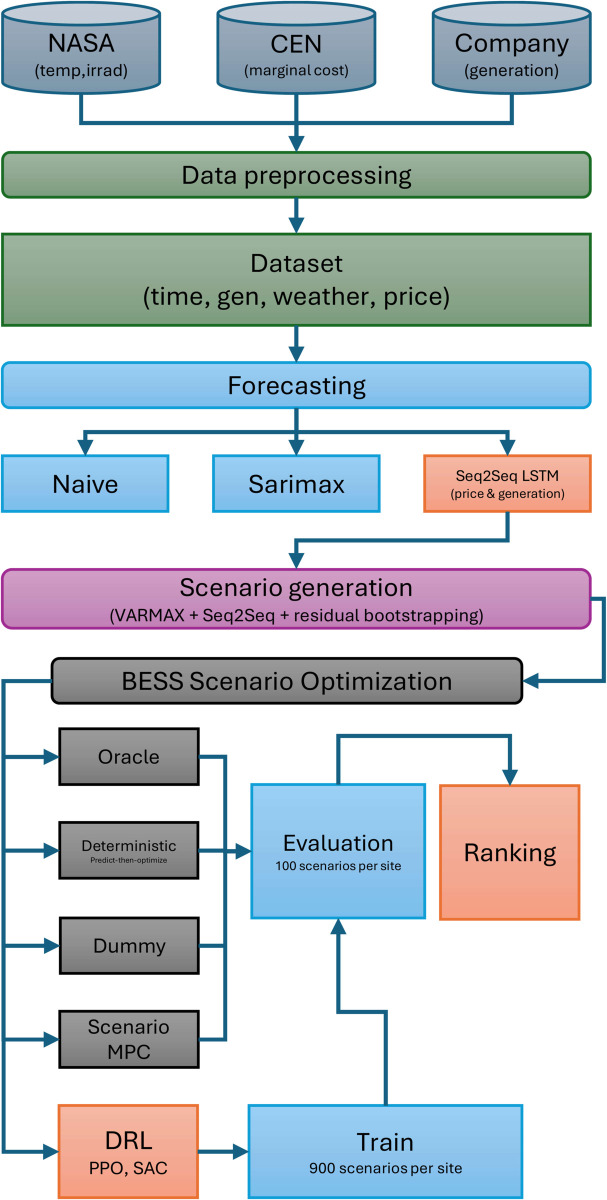
Overall methodological workflow. Historical data from NASA POWER, the Chilean system operator (CEN), and the plant operator are preprocessed into a unified dataset. Three forecasting models (Naive, SARIMAX, Seq2Seq LSTM) are trained, and the Seq2Seq model is used together with VARMAX and residual bootstrapping to generate price and generation scenarios. These scenarios feed the BESS scenario-optimization layer, which evaluates four control strategies (Oracle, deterministic predict-then-optimize, Scenario MPC, and DRL agents; PPO and SAC) plus a Dummy baseline. DRL agents are trained on 900 scenarios per site and evaluated on 100 held-out scenarios, and all controllers are ranked based on their out-of-sample performance.

Fig 1 the first stage is the collection and preprocessing of historical data, which provides the empirical foundation for the subsequent forecasting stage. In this second stage, we develop and train the predictive models (SARIMAX and LSTM) described in Sect 3.2; additionally, as a baseline for comparison, we include a simple Naive estimation scheme. As an early preview, the LSTM-Seq2Seq model outperforms the others and is then enhanced by generating a large set of stochastic scenarios designed to capture the inherent uncertainty of market prices and PV generation. This set of scenarios forms the basis for the core of our study: a comparative evaluation of four distinct BESS control strategies. These strategies range from a theoretically optimal Oracle with perfect foresight and a baseline Dummy agent with a random policy to two practical approaches: a predict-then-optimize scheme serving as the main benchmark, and our proposed DRL agents trained on the full distribution of scenarios. Finally, the economic performance of each strategy is evaluated and compared to quantify the value added by the data-driven DRL approach.

Our methodology is structured as a comprehensive multistage pipeline designed to develop, validate, and benchmark a data-driven control strategy for a PV–BESS system. The overall workflow is summarized in Fig 1. Historical meteorological, price, and generation data (NASA POWER, CEN, and plant operator) are preprocessed into a unified dataset. Three forecasting models (Naive, SARIMAX, and a Seq2Seq LSTM) are trained, and the Seq2Seq model is then used, together with a VARMAX weather model and residual bootstrapping, to generate large ensembles of joint price–generation scenarios. This common scenario set feeds four control approaches: (i) an Oracle with perfect foresight, (ii) a deterministic predict–then–optimize LP, (iii) a scenario-based MPC controller, and (iv) DRL agents (PPO and SAC) trained offline and evaluated online, plus a Dummy baseline. The diagram explicitly highlights how the Seq2Seq forecaster and DRL controllers are integrated into a single forecast-and-control pipeline.

### 3.1 Data collection and preprocessing

For this study, we constructed an hourly data set that spans two full years (2022-2023) for three commercial-scale PV plants located in northern Chile: Cachiyuyo, Illapel, and Romeral. The dataset integrates information from three distinct sources:

1. *Meteorological data:* Historical hourly weather data was obtained from the NASA Prediction of Worldwide Energy Resources (POWER) project [42]. For the specific coordinates of each PV plant, we gathered key variables known to influence photovoltaic performance: Global Horizontal Irradiance (GHI) in W/m^2^ and air temperature at 2 meters in ^∘^C. This dataset provides a consistent and scientifically validated source for the primary drivers of PV generation.
2. *Nodal price data:* The hourly marginal prices for the corresponding electrical nodes of each plant were obtained from the public database of the Chilean National Electric Coordinator (CEN), which is the country’s independent system operator. The nodes used were “Diego de Almagro” for the Cachiyuyo plant, “Los Vilos 220kV” for Illapel, and “Copiapo 110kV” for Romeral. These prices represent the real economic signals to which the BESS must respond.
3. *Generation data:* Actual energy generation data (in MWh) was provided by the plant operator. These ground truth data reflect the real-world production of the plants, accounting for all operational factors, including efficiency losses, curtailment events, and maintenance.

We merged these data into a single hourly time-series dataset containing, per PV-BESS: date, weather (GHI/TEMP), price, and generation. The preprocessing steps included the removal of duplicate timestamps and the treatment of a few missing values (less than 1%), mainly in the weather variables, using forward-fill imputation when necessary. This process produced a clean and consistent dataset, ready to be used in the forecasting and DRL stages.

### 3.2 Forecasting models for price and generation

The PV-BESS control strategy requires an accurate forecast of nodal prices and generation. To provide these inputs to our DRL agents, we developed and compared three time-series forecasting models: a seasonal Naive benchmark, a SARIMAX/ARIMA model, and an LSTM–Seq2Seq architecture.

The forecasting methodology has two independent but analogous parts, one for solar generation and the other for electricity prices. The objective of this approach is to generate forecasts to be used as inputs for a DRL based BESS control agent. For each variable, we trained three models and evaluated them to identify the better approach. These models include a simple seasonal benchmark (Naive), a standard statistical model (SARIMAX), and an LSTM-Seq2Seq.

#### 3.2.1 Seasonal naive model.

This model serves as a robust baseline to evaluate the performance of more complex models. It operates on the assumption that the value at a specific hour *t* is equal to the value observed at the same hour on the previous day, i.e., at time *t* – 24. Its straightforward mathematical formulation is presented in Eq 1.

$${\hat{y}}_{t}=y_{t-24}$$ (1)

where ${\hat{y}}_{t}$ is the forecast for hour *t*, and *y*_*t*−24_ is the observed value 24 hours before.

#### 3.2.2 SARIMAX model.

The Seasonal Autoregressive Integrated Moving Average with eXogenous variables (SARIMAX) model was implemented as a standard statistical benchmark. For the solar generation forecast, the model incorporated Global Horizontal Irradiance (GHI), ambient temperature, and time-based cyclical features as exogenous variables (*X*_*t*_). For the electricity price forecast, only the cyclical features based on time were included as external input. To ensure robustness and avoid potential instability caused by hyperparameter tuning, we adopted a simple yet effective model order of SARIMAX (1,1,0)(1,0,1)_24_ for both forecast pipelines. The implementation relied on an off-the-shelf library employing the auto_arima procedure proposed by [43].

#### 3.2.3 Sequence-to-sequence (Seq2Seq) model.

A sequence-to-sequence architecture (Seq2Seq) with long-short-term memory cells (LSTM) was selected as the primary forecasting model. This architecture is adequate for sequence prediction tasks. It comprises two main components:

- **Encoder:** An LSTM network that processes an input sequence of the last 24 hours of all relevant features. It compresses this historical information into a fixed-size context vector, which consists of the final hidden and cell states of the LSTM.
- **Decoder:** Another LSTM network that is initialized with the context vector of the encoder. The forecast for the next 24 hours is then generated step by step, where the output of each step can be used as input for the subsequent one.

**Fig 2:**
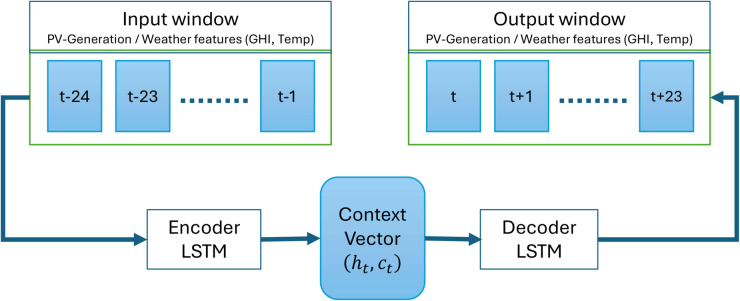
Schematic of the Seq2Seq LSTM forecaster. The encoder processes the last 24 hours of multivariate historical features (generation or price, exogenous variables, and time encodings) and compresses them into a context vector. The decoder is initialized with this context vector and generates the 24-hour-ahead forecast sequentially, one hour at a time. The same architecture is used for both the price and generation forecasting models.

Fig 2 illustrates the Seq2Seq LSTM architecture used for both price and generation forecasting. The encoder consumes a 24-hour window of historical features and compresses them into a context vector, which initializes the decoder that recursively produces the 24-step-ahead forecasts.

For price and generation, the Seq2Seq model is trained as a standard point forecaster. In the scenario-generation stage (Sect 3.3) we treat its one-step-ahead predictions as conditional means and construct probabilistic trajectories around them via residual bootstrapping. This distinction is important: the predict-then-optimize baseline uses the Seq2Seq point forecasts directly, whereas the DRL agents and the Scenario MPC controller consume the entire scenario ensemble.

### 3.3 Scenario generation

To provide DRL agents with the necessary probabilistic forecasts, a scenario generation process was developed. This process is detailed below:

1. *Weather Scenarios:* A multivariate Vector Autoregressive Moving Average with eXogenous variables (VARMAX) model was trained on historical GHI and temperature data. Then, this model was used to generate 1,000 correlated weather scenarios for the forecast horizon.
2. *Generation Scenarios:* The trained Seq2Seq generation model used each of the 1,000 weather scenarios as input. This produced 1,000 corresponding solar generation scenarios. To account for the uncertainty of the model, residual bootstrapping was applied to the predictions.
3. *Price Scenarios:* Since the Seq2Seq price model is independent of weather variables, it generated 1,000 price scenarios recursively. The uncertainty of the model was also incorporated into these scenarios via the residual bootstrapping technique.

#### 3.3.1 Regulatory context (Chile).

It is important to clarify that the three sites in this study operate under the Chilean *Pequeños Medios de Generación Distribuida* (PMGD) regime established by Supreme Decree No. 88 (*Decreto Supremo* 88). With installed capacity below the 9 MW threshold defined in Article 2, these units are eligible to operate outside the centralized unit commitment economic dispatch process of the national system operator (*Coordinador Eléctrico Nacional*) and to inject energy and be remunerated under the *stabilized price* mechanism set out in Articles 7 and 8. Consistent with this regulatory setting, our modeling treats nodal prices as exogenous signals and does not include market clearing or bidding behavior.

The Chilean PMGD regime is not unique; some other countries share similar policies that promote decentralized generation [44]. These frameworks share some common goals, such as minimizing investment risk and guaranteeing market access for smaller energy assets [45]. Some relevant examples are the Feed-in Tariffs (FiT) pioneered by Germany’s Renewable Energy Sources Act (EEG) and the *avoided cost* remuneration under the U.S. Public Utility Regulatory Policies Act (PURPA). This global context underscores the broader relevance of our price-based self-scheduling problem, as asset owners face similar economic and operational challenges across many jurisdictions.

### 3.4 Plant-level price-based self-scheduling problem

We study the plant-level, price-based self-scheduling of a grid-connected photovoltaic (PV) plant co-located with a battery energy storage system (BESS). At each hour, the controller decides how much PV energy to inject into the grid and how much to store in the BESS to maximize revenue given the *exogenous* nodal prices. No market clearing, bidding behavior, or network constraints are modeled; only device physics and operational limits are enforced.

#### 3.4.1 Technical parameters of the PV-BESS system.

Across the three sites (Cachiyuyo, Illapel and Romeral) we standardize the BESS to a six-hour device with inverter limit $P^{\max}=4$ MW and nominal energy $E^{nom}=24$ MWh, as summarized in Table 2. The round trip efficiency is fixed at 95% (we use symmetric charging and discharging efficiencies so that $\eta_{c}\eta_{d}=0.95$), and the state of charge (SoC) is restricted to [20%,90%] of $E^{nom}$. Grid charging is disabled; the battery can only be charged from PV. These values are enforced both in the optimization baselines and in the DRL environment.

**Table 2:** BESS parameters used in all experiments.

Inverter power limit	$P^{\max}$ = 4 MW
Nominal energy	$E^{nom}=24$ MWh
Round-trip efficiency	$\eta_{c}\eta_{d}=0.95$
Operational SoC range	$[0.20,\;0.90]\cdot E^{nom}$
Time step	Δt=1 hour

#### 3.4.2 Plant-level formulation or predict–then–optimize approach.

Let the discrete-time index t=0,…,T−1 denote the hourly steps within the optimization horizon. The model parameters include the available PV generation *G*_*t*_ (MWh), the nodal energy price *p*_*t*_ ($/MWh), and the initial state of charge, *s*_0_. The decision variables, representing energy flows in MWh per hour, are:

- $E_{t}^{pv\to grid}\geq 0$: Energy injected directly from the PV plant to the grid.
- $E_{t}^{pv\to bess}\geq 0$: Energy used to charge the BESS from the PV plant.
- $E_{t}^{bess\to grid}\geq 0$: Energy discharged from the BESS to the grid.
- $s_{t}\in [s_{\min},s_{\max}]$: State-of-charge variables.

The objective is to maximize the total revenue from energy sales over the horizon, as formulated in (2):

$$\max\sum_{t=0}^{T-1}\left(E_{t}^{pv\to grid}+E_{t}^{bess\to grid}\right)\;p_{t}.$$ (2)

This optimization is governed by a set of physical and operational constraints:

$$E_{t}^{pv\to grid}+E_{t}^{pv\to bess}\leq G_{t},\;\;\;\;\forall t,$$ (3)

$$s_{t+1}=s_{t}+\eta_{c}E_{t}^{pv\to bess}-\frac{1}{\eta_{d}}E_{t}^{bess\to grid},\;\;\;\;\forall t,$$ (4)

$$E_{t}^{pv\to bess}\leq P^{\max}\Delta t,\;E_{t}^{bess\to grid}\leq P^{\max}\Delta t,\;\;\;\;\forall t,$$ (5)

The constraints ensure the physical integrity of the system’s operation. Specifically:

- *PV Energy Balance (3)*: Ensures that the total energy injected from the PV plant (either to the grid or to the BESS) does not exceed the available generation *G*_*t*_ in each hour.
- *SoC Dynamics (4)*: Models the evolution of the SoC, where the SoC at the next step (*s*_*t* + 1_) is determined by the previous state (*s*_*t*_), the energy charged, and the energy discharged, adjusted by their respective efficiencies ($\eta_{c}$ and $\eta_{d}$).
- *Power Limits (5)*: Restricts the charging and discharging rates to the maximum power capacity of the inverter, $P^{\max}$, during the time step Δt.

This linear programming (LP) formulation defines the stage-wise revenue and device dynamics that underlie all optimization-based baselines. The Oracle and the deterministic predict–then–optimize benchmark solve (2)-(5) directly, using either perfect foresight or a point forecast for (*G*_*t*_,*p*_*t*_), and the Scenario MPC controller extends the same model to a case with multiple scenarios, receding-horizon setting. In contrast, the DRL and Dummy agents interact with the PV–BESS system through a Markov decision process built on the same SoC dynamics and revenue definition, as described below.

### 3.5 Markov decision process (MDP) formulation for DRL control

The interaction between the DRL agents and the PV-BESS system is modeled as a finite-horizon MDP with horizon *T* = 336 steps (14 days), consistent with the optimization model in (2)-(5). The MDP is defined by the tuple (𝒮,𝒜,P,r,γ) as follows.

**State.** Let $E_{\text{nom}}$ denote the nominal energy capacity of the BESS (MWh). We work with a normalized state of charge


$$q_{t}=\frac{s_{t}}{E_{\text{nom}}}\in [q_{\min},q_{\max}],$$

where *s*_*t*_ is the SoC variable in (6) and $q_{\min},q_{\max}$ are the operational SoC limits (for example, 0.20 and 0.90). In addition to *q*_*t*_, the agent observes summaries of the forecast information and calendar time. Specifically, at each hour *t* we compute the mean price ${\bar{p}}_{t}$ and mean PV generation ${\bar{G}}_{t}$ across the forecast scenario ensemble, together with a vector of cyclical time features


$$\phi_{t}=(\sin(2\pi {hour}_{t}/24),\cos(2\pi {hour}_{t}/24),\sin(2\pi {dow}_{t}/7),\cos(2\pi {dow}_{t}/7)).$$

The MDP state is then $x_{t}=(q_{t},{\bar{p}}_{t},{\bar{G}}_{t},\phi_{t})\in 𝒮$.

**Action.**

The action space is one-dimensional and continuous,


𝒜=[−1,1].

At each hour, the agent selects $a_{t}\in [-1,1]$, which is assigned to candidate charging and discharging powers relative to the inverter limit $P_{\max}$:


$$P_{t}^{ch}=P_{\max}\;\max(-a_{t},0),\;P_{t}^{dis}=P_{\max}\;\max(a_{t},0).$$

With a time step Δt=1 hour, the corresponding energy flows are


$$E_{t}^{ch}=P_{t}^{ch}\Delta t,\;E_{t}^{dis}=P_{t}^{dis}\Delta t.$$

The environment then enforces feasibility by truncating these flows so that they respect the PV availability, power limits, and SoC bounds implied by constraints (3)-(5). In particular, simultaneous charging and discharging is prevented, and any infeasible suggestion is projected onto the admissible set.

**Transition dynamics.**

The only controlled state variable is the SoC. Given the realized PV generation and the price at time *t* for a particular test scenario, denoted $G_{t}\text{and}p_{t}$, the feasible flows $E_{pv\to bess,t}$ and $E_{bess\to grid,t}$ are derived from ($E_{t}^{ch},E_{t}^{dis}$) and *G*_*t*_ under (3)–(5). The SoC then evolves according to the same physical model used in the LP,

$$s_{t+1}=s_{t}+\eta_{c}E_{pv\to bess,t}-\frac{1}{\eta_{d}}E_{bess\to grid,t},$$ (6)

with *s*_*t* + 1_ clipped to $\left[s_{\min},s_{\max}\right]$ to enforce the SoC limits. The normalized SoC *q*_*t* + 1_ is obtained from *s*_*t* + 1_ as above, and the forecast summaries $\left({\bar{p}}_{t+1},{\bar{G}}_{t+1}\right)$ and time features $\phi_{t+1}$ are updated deterministically from the scenario ensemble and calendar. Price and generation trajectories are exogenous; they enter the transition kernel $P(\cdot |x_{t},a_{t})$ through the realised scenario paths.

**Reward.** The instantaneous reward is the revenue from energy sold to the grid in hour *t*, consistent with the revenue objective (2),


$$r_{t}=p_{t}\left(E_{pv\to grid,t}+E_{bess\to grid,t}\right),$$

where $E_{pv\to grid,t}$ and $E_{bess\to grid,t}$ are the physically truncated flows resulting from the chosen action *a*_*t*_ and the realized PV generation *G*_*t*_.

**Objective.** The DRL agents seek a policy $\pi (a_{t}|x_{t})$ that maximizes the expected discounted return over the 14-day horizon,


$$J(\pi )=𝔼\;\left[\sum_{t=0}^{T-1}\gamma^{t}r_{t}\right],$$

where the expectation is taken over the stochastic price and generation paths and, when relevant, over the policy-induced randomness. We use a discount factor γ=0.99.

In this formulation only the DRL agents directly optimize the expectation J(π). Scenario MPC optimizes, at each hour, the sample-average revenue over the *K* training scenarios in its look-ahead window, which is an empirical approximation of an expected value. In contrast, the Oracle and deterministic predict–then–optimize baselines solve deterministic LPs for each scenario or for a point forecast, and the Dummy baseline does not optimize an expectation at all. All methods are evaluated ex post using the mean profit across the 100 test scenarios.

The Oracle, deterministic predict-then-optimize, and Scenario MPC baselines can be interpreted as open-loop or receding-horizon solutions for the same underlying system, while the DRL policies implement a closed-loop mapping $x_{t}\mapsto a_{t}$ that adapts hourly to the realized trajectory.

### 3.6 Scenario-based model predictive control or Scenario MPC

We include a scenario-based model predictive control (Scenario MPC) baseline as a stochastic optimization counterpart to the deterministic predict-then-optimize strategy. This controller approximates multi-stage stochastic programming using the same price-generation scenario ensemble employed to train the DRL agents. At each hour *t* of the 14–day horizon (*T* = 336 steps), it solves a short-horizon linear program over a subset of training scenarios and applies only the first-step decision in a receding-horizon fashion.

Let 𝒦 denote a subset of *K* = 30 scenarios sampled from the 900 training trajectories used for DRL, and let *H* = 24 be the look-ahead window in hours. For a given site and time *t* we build a block of prices and PV generation $\{p_{\tau }^{\left(k\right)},G_{\tau }^{\left(k\right)}\}\text{for}\tau =t,\ldots ,t+H_{eff}-1$ and k∈𝒦, where


$$H_{eff}=\min(H,T-t).$$

For each scenario *k* and step τ we use the same energy-flow variables as in the linear programming model (LP), $E_{pv\to grid,\tau }^{\left(k\right)}$, $E_{pv\to bess,\tau }^{\left(k\right)}$, and $E_{bess\to grid,\tau }^{\left(k\right)}$, and enforce the device physics independently per scenario (PV balance, power limits, SoC dynamics, and SoC bounds).

The Scenario MPC optimization problem at time *t* maximizes the sample-average revenue over the look-ahead window,

$$\max\;\frac{1}{K}\sum_{k\in 𝒦}\sum_{\tau =t}^{t+H_{eff}-1}p_{\tau }^{\left(k\right)}\left(E_{pv\to grid,\tau }^{\left(k\right)}+E_{bess\to grid,\tau }^{\left(k\right)}\right),$$ (7)

subject to the device constraints (3)-(5) applied to each scenario and time step, with the initial state of charge at *t* shared across all scenarios. Non-anticipativity is enforced only at the current hour: the first-step BESS charge and discharge flows, $E_{pv\to bess,t}^{\left(k\right)}$ and $E_{bess\to grid,t}^{\left(k\right)}$, are constrained to be identical for all k∈𝒦. Future steps within the horizon are scenario specific and serve only as a look-ahead to approximate the value of current decisions.

We set the look-ahead window to *H* = 24 hours and use *K* = 30 training scenarios per site in the final experiments. The optimization at each hour is solved with the HiGHS backend, and the common first-step BESS charge and discharge decisions are applied to the true test scenario, updating the SoC according to (6). Repeating this receding-horizon procedure for all *T* = 336 hours and for each of the 100 test scenarios yields a Scenario MPC policy that is directly comparable with the DRL agents and the other baselines. This configuration (*H* = 24, *K* = 30) is deliberately modest: it reflects a tractable, practitioner-oriented implementation that balances the representation of uncertainty with the computational cost of solving a multi-scenario LP every hour, rather than an idealized multi-stage stochastic program.

### 3.7 Solution methodologies

We compare the following approaches:

- *Oracle (perfect foresight):* For each scenario we solve (2)–(5) with the true future *G*_*t*_ and *p*_*t*_. This benchmark is unreachable in practice and serves as an upper bound and a strong check against data leakage.
- *Deterministic optimizer or predict-then-optimize:* We compute a single open-loop operating plan by solving the LP with objective (2) subject to the device constraints (3)-(5) on an aggregate forecast (empirical mean across scenarios). The aggregate forecast is built from the Seq2Seq model in Sect 3.2 by taking, at each hour, the mean across the 900 training scenarios generated as described in Sect 3.3. The resulting plan is then applied to each test scenario with physical truncation at runtime so that the realized flows always satisfy (3)-(5). This baseline emulates an engineer using point forecasts from the Seq2Seq model to precommit an operating plan; it does not optimize an expectation over scenarios, although the reported performance is always summarized as average profit across the 100 test scenarios.
- *Scenario-based MPC (Scenario MPC):* A scenario-based receding-horizon controller that, at each hour, solves the short-horizon multi-scenario LP in (7) using a subset of the training ensemble and applies only the first-step BESS decision, thereby approximating a stochastic programming solution in a computationally tractable way.
- *DRL agents (PPO and SAC):* We train two Deep Reinforcement Learning agents, Proximal Policy Optimization (PPO) and Soft Actor-Critic (SAC), on a custom Gymnasium environment that encapsulates the same physics and limits described above. Observations include the current SoC fraction, the mean price and mean generation at time *t* across the forecast ensemble, and compact time encodings (sine/cosine of hour and day of week). The action is a scalar $a_{t}\in [-1,1]$ assigned to a feasible charge/discharge in kWh per hour. We use VecNormalize to standardize observations (and reward for PPO during training), keep the evaluation in physical units, and select the champion per site/agent as the model with the best evaluation return. Three network capacities are explored (small, medium, and large), and the best is retained for testing.
- *Dummy:* A feasibility-respecting baseline that draws, at every hour, a uniform action within the admissible charge/discharge limits implied by the current SoC, the inverter limit and the available photovoltaic. It is intentionally simple and nonrandom with respect to physics limits; it provides a lower bound to quantify the improvement over nonoptimized behavior.

## 4 Results and analysis

### 4.1 Generation and price forecast results

The models were trained on one year of historical data and subsequently evaluated on a 14-day hold-out set. Performance was quantified using the Root Mean Square Error (RMSE) and the Mean Absolute Error (MAE). The results for both generation and price forecasts are summarized in Table 3.

**Table 3:** Forecast performance for generation and price across sites.

Site	Model	Generation		Price	
		RMSE	MAE	RMSE	MAE
Cachiyuyo	Naive	2.44	1.52	40.38	18.81
Cachiyuyo	SARIMAX	3.47	3.00	40.29	29.39
Cachiyuyo	Seq2Seq	1.53	0.93	28.32	15.26
Illapel	Naive	1.05	0.68	40.52	18.54
Illapel	SARIMAX	0.88	0.56	41.93	30.10
Illapel	Seq2Seq	0.60	0.38	26.41	15.38
Romeral	Naive	2.55	1.55	41.65	19.29
Romeral	SARIMAX	2.00	1.19	43.12	31.61
Romeral	Seq2Seq	1.65	0.98	27.27	17.43

The results consistently show the superiority of the Seq2Seq model across all sites for both forecasting tasks. This validates its selection to generate the scenarios used in the subsequent DRL training phase.

**Fig 3:**
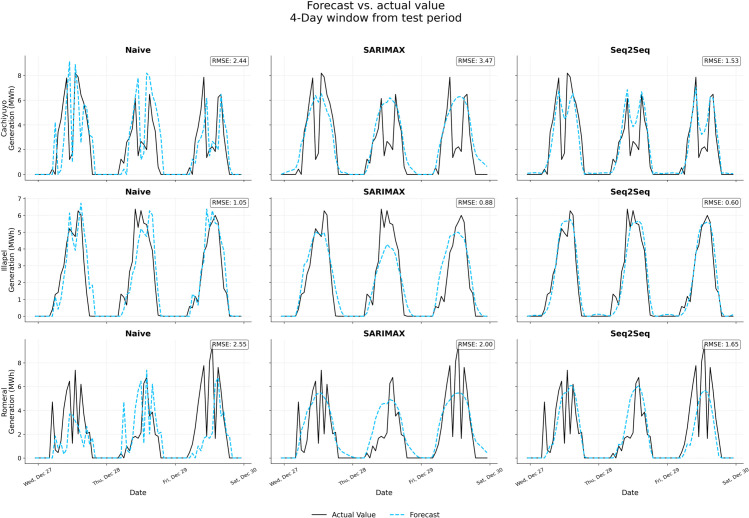
Generation forecast results for a 4-day sample period.

Fig 3 reports a representative 4-day window for generation forecasts; the analogous price forecast panel is provided in Supporting Information (S1 Fig).

### 4.2 DRL agents training and performance

#### 4.2.1 Agent capacities and main hyperparameters.

Both PPO and SAC use feed-forward multi-layer perceptron (MLP) networks for the policy and value functions. We explore three network-capacity tiers (Small/Medium/Large), summarized in Appendix B, Table 1, and select the best tier per site and algorithm.

Unless otherwise stated, we keep the default hyperparameters of stable-baselines3. Here we list the main settings that are relevant for reproducibility:

- *Episode length and discounting:* each episode spans *T* = 336 hourly steps (14 *days*) with discount factor γ=0.99.
- *Network capacities:* for PPO we use separate MLPs for the policy and value function with the layer sizes listed in Table 1 (Small, Medium, Large); for SAC we use a shared MLP with the same three capacity tiers.
- *PPO-specific settings:* clipping range 0.2, generalized advantage estimation parameter λ=0.95, value-loss coefficient 0.5, and gradient-norm clipping at 0.5.
- *SAC-specific settings:* automatic entropy-temperature tuning (ent_coef="auto") and soft target-network updates with τ=0.005.
- *Normalization and early stopping:* we use VecNormalize to normalize observations (and rewards for PPO during training) and an EvalCallback combined with StopTrainingOnNoModelImprovement to implement evaluation-driven early stopping and select the champion model per site.

For ease of reference, a complete list of abbreviations used throughout the paper is provided in Appendix C (Table 1).

All models are trained on 900 scenarios per site and evaluated on a held-out set of 100 scenarios, as detailed in the next subsection.

#### 4.2.2 Experimental design and evaluation protocol.

We work with scenario ensembles for both price and generation at each site. Each CSV contains an hourly datetime index and columns prediction_1, prediction_2, . We slice a 14-day horizon (*T* = 336 hours) and adopt the following split: the first 900 scenarios are used for training and model selection, while the last 100 scenarios are reserved for testing. After selecting the per-site champion for PPO and SAC, we evaluated them on the 100 test scenarios, together with the Oracle, Deterministic, and Dummy baselines. A summary of the selected champion DRL agents per site, including their mean profits, is provided in Appendix B (Table 1).

Our primary metric is the total profit (USD) per scenario. We also report operational metrics: total charged/discharged energy, equivalent full cycles $=\frac{\sum_{t}E_{t}^{bess\to grid}\;[kWh]}{E^{nom}[kWh]}$, and two contracyclical indicators: Pearson correlation between price and median SoC, and the fraction of hours with (low price and high SoC) or (high price and low SoC).

#### 4.2.3 Training stability and computational cost.

**Fig 4:**
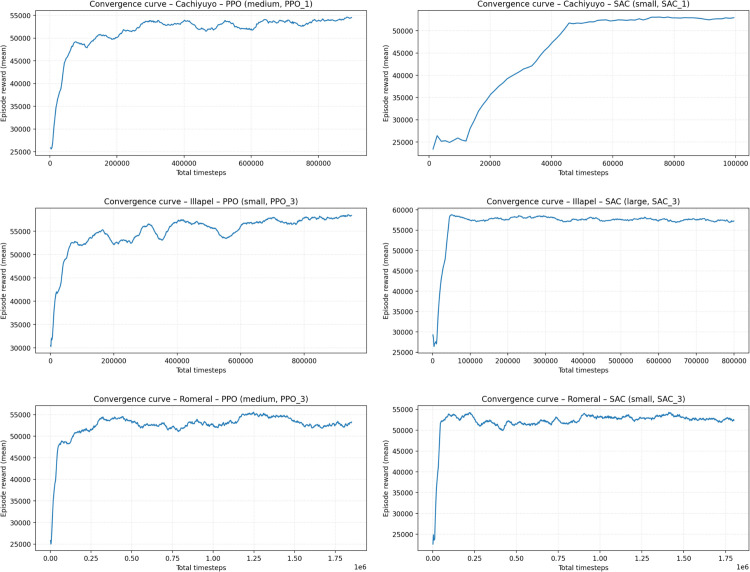
Learning curves (mean episode reward vs. total timesteps) for the final PPO and SAC agents used in the evaluation at each site. Plots correspond respectively to Cachiyuyo—PPO (medium), Cachiyuyo—SAC (small), Illapel—PPO (small), Illapel—SAC (large), Romeral—PPO (medium), and Romeral—SAC (small).

To assess training stability and provide a transparent view of the computational effort, Fig 4 reports the learning curves (mean episode reward vs. total timesteps) for the final PPO and SAC agents used in the evaluation at each site. In all cases, the mean return increases rapidly during the initial $O(10^{4}\;-\;10^{5})$ steps and then stabilizes, with only mild oscillations, which is consistent with the use of evaluation-driven early stopping and the regularization choices described above (policy clipping, entropy regularization, gradient clipping, and soft target updates). This behavior indicates that the selected agents have reached a stable performance plateau instead of relying on a transient peak.

Table 4 summarizes the corresponding wall-clock training times on the NVIDIA RTX 4090 workstation. Training steps range from roughly $1\times 10^{5}$ to $1.8\times 10^{6}$ transitions, with wall-clock times between about 0.2 h and 4.2 h per agent, depending on the algorithm and network size. The last column reports the final mean episode reward taken from the TensorBoard logs, which is consistent with the profits reported in the main results. Overall, these figures confirm that the proposed DRL training setup is computationally tractable on a single modern graphics processing unit (GPU) and yields stably convergent policies for all three plants.

**Table 4:** Wall-clock training time and convergence statistics for the DRL agents used in the evaluation. For each site and algorithm we report the selected network capacity, total number of training steps, approximate wall-clock training time on the NVIDIA RTX 4090 workstation, and the last recorded value of the mean episode reward from the TensorBoard logs.

Site	Agent	Network size	Training steps	Training time [h]	Final mean episode reward
Cachiyuyo	PPO	Medium	899,072	0.28	54,428
Cachiyuyo	SAC	Small	99,456	0.19	52,126
Illapel	PPO	Small	949,248	0.32	58,545
Illapel	SAC	Large	799,680	1.84	59,569
Romeral	PPO	Medium	1,849,344	0.62	53,387
Romeral	SAC	Small	1,799,616	4.20	54,033

#### 4.2.4 Results.

**Fig 5:**
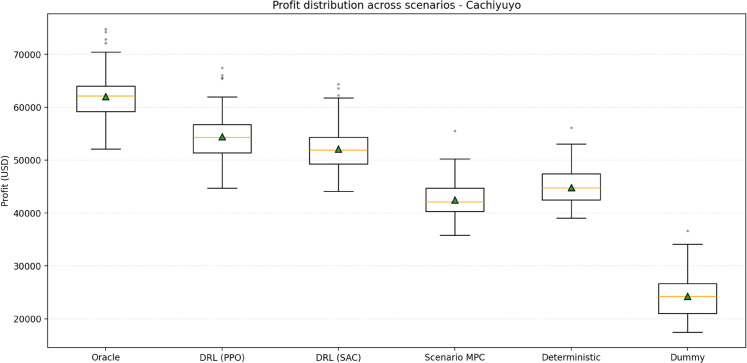
Profit distribution across scenarios - Cachiyuyo.

Fig 5 illustrates the profit distribution for Cachiyuyo. The corresponding distributions for Illapel and Romeral are reported in the Supporting Information (S7 Fig and S8 Fig). The ordering with respect to performance is consistent with expectations: the Oracle provides the upper bound, followed by the DRL agents, the model-based strategies, and finally the Dummy policy. Both PPO and SAC clearly dominate the alternatives; while their performance is nearly identical—alternating slightly by site—they achieve the highest average profit while preserving moderate cycling, confirming their ability to leverage the full scenario distribution effectively.

Among the model-based baselines, the deterministic predict-then-optimize LP and the Scenario MPC controller deliver similar but not identical profits. Scenario MPC, which explicitly accounts for stochasticity through a 24-hour look-ahead window and a subset of *K* = 30 training scenarios, lies slightly below the deterministic LP in mean profit across sites. This reflects its conservative design: by optimizing the average revenue over a small scenario set and enforcing a common first-step decision, the controller sacrifices some opportunity in high-spread situations in exchange for robustness. Under the horizon length and sample size used here, this leads to realized performance that is close to, but marginally worse than, the deterministic plan built on a single aggregate forecast.

**Fig 6:**
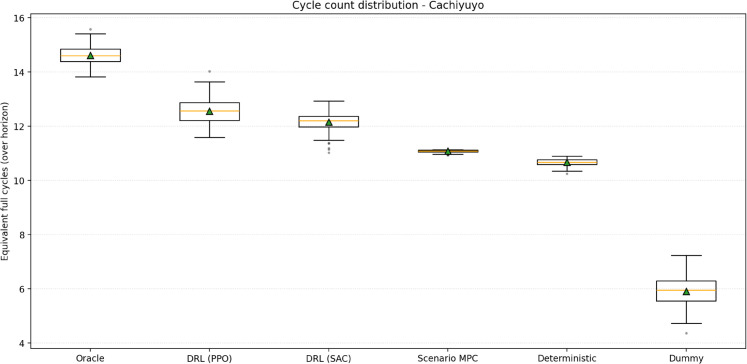
Equivalent full cycles over the horizon - Cachiyuyo.

The equivalent full-cycle distributions are reported in Fig 6 for Cachiyuyo; the corresponding results for Illapel and Romeral are presented in the Supporting Information (S9 Fig and S10 Fig). As expected, the Oracle executes the largest number of cycles, with the DRL agents slightly below it and above the deterministic and Scenario MPC baselines. Scenario MPC performs a very similar number of equivalent full cycles to the deterministic LP—slightly higher in Cachiyuyo—yet obtains lower profits. This indicates that the gap between these two controllers is driven mainly by the timing of charge/discharge decisions rather than by how intensively the battery is used. The narrow dispersion of cycle counts for Scenario MPC across test scenarios is consistent with its receding-horizon design, which uses the same subset of training trajectories at each hour and therefore produces very similar operating patterns across the test ensemble.

**Fig 7:**
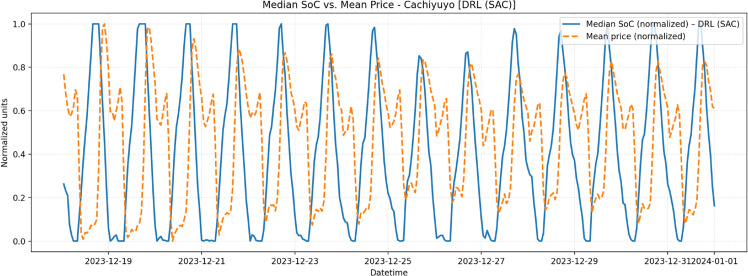
Median SoC vs. mean price (normalized) for Cachiyuyo with agent SAC.

For Cachiyuyo, the SoC–price overlay obtained with the SAC agent is shown in Fig 7. The corresponding PPO result and the overlays for Illapel and Romeral are given in the Supporting Information (S1 Appendix; see S2 Fig and S3–S6 Figs). All profiles exhibit the expected *contracyclical* behavior: the SoC tends to be higher during low-price hours and lower during high-price hours, consistent with the negative hour-by-hour SoC–price correlations computed from the scenario medians.

#### 4.2.5 Analysis.

The comparison confirms our central hypothesis regarding the practical advantages of adaptive DRL policies over fixed, deterministic plans in uncertain operating environments. First, the DRL agents, trained on a diverse ensemble of probabilistic scenarios, learn adaptive policies that inherently account for forecast uncertainty. This contrasts sharply with the predict-then-optimize baseline, which commits to a single operational plan based on an average forecast; a future that rarely materializes exactly. Consequently, the DRL agents consistently outperform this traditional, more brittle approach, highlighting the limitations of deterministic optimization paradigms for real-time control under uncertainty. While stochastic programming could theoretically model uncertainty, its computational burden is often prohibitive for high-frequency decision-making [19]. Our DRL framework offers a computationally tractable alternative, learning a robust, near-optimal policy offline that enables fast, adaptive online decisions. Second, the performance gap relative to the perfect-foresight Oracle is consistent and reasonable, indicating effective learning without data leakage, as agents respond appropriately to the realistic uncertainty within the scenarios. Furthermore, the narrower profit interquartile ranges observed for DRL agents in several cases (Fig 5) suggest greater policy robustness compared to the fixed baseline, reflecting the benefit of hourly adaptation versus adherence to a pre-committed plan.

It is also crucial to compare our approach against optimization-based models that handle stochasticity explicitly. We developed a scenario-based MPC that accounts for price and generation uncertainty using the same scenario ensemble as the DRL agents. This controller approximates a multi-stage stochastic program by solving, at each hour, a short-horizon multi-scenario LP with non-anticipative first-step decisions, under a pragmatic configuration of *H* = 24 and *K* = 30.

Empirically, Scenario MPC achieves intermediate performance: it clearly outperforms the Dummy policy but remains slightly below the deterministic predict-then-optimize baseline, despite executing a similar number of equivalent full cycles. This behaviour is consistent with a conservative controller that optimizes the sample-average revenue over a small training ensemble, leading to robust but somewhat myopic decisions. Under these realistic computational constraints, stochastic optimization does not provide a decisive advantage over deterministic planning, whereas DRL leverages the full distribution of scenarios offline to deploy fast and adaptive policies online.

The differences observed between PPO and SAC are not statistically or empirically conclusive. Both achieve equivalent performance within expected variance, suggesting that the decisive factor was the quality of the forecasting and scenario ensemble rather than the specific actor–critic algorithm employed.

#### 4.2.6 Computational setup and software.

All experiments were performed on a Windows workstation (Intel Core i9-13900KF, 64 GB RAM, NVIDIA GeForce RTX 4090 with 24 GB VRAM). The environment was implemented in Python with Gymnasium and Stable-Baselines3; linear programs were solved with the highs backend. We used GPU acceleration for DRL training. Reproducible scripts for the environment, training (including VecNormalize and evaluation callbacks) and baselines are available in the project code base.

#### 4.2.7 Interpretation of DRL and baseline performance.

The comparison confirms our central goal of showing evidence of the practical advantages of adaptive DRL policies over deterministic plans in uncertain operating environments. First, DRL agents, trained in probabilistic scenarios, learn adaptive policies that inherently account for the uncertainty of the forecast. This contrasts with the predict-then-optimize baseline, which follows a single operational plan based on an average forecast; a future that rarely occurs. Consequently, DRL agents outperform the traditional optimization approach and highlight its limitations for real-time control under uncertainty.

Other optimization approaches that include a more realistic representation of uncertainty are stochastic programming and scenario-based MPC. Nevertheless, fully fledged stochastic programs are prohibitive for high-frequency decision making [19], and even our pragmatic Scenario MPC configuration—with a 24-hour look-ahead and *K* = 30 training scenarios—did not outperform the deterministic predict-then-optimize benchmark: it achieved slightly lower profits while using a similar number of equivalent full cycles. This behaviour is consistent with a conservative controller that optimizes sample-average revenue over a small ensemble. In contrast, our DRL approach offers a computationally tractable alternative, learning a robust, near-optimal policy that allows for fast, adaptive online decisions. Second, the performance gap relative to the perfect-foresight Oracle is consistent and reasonable, indicating effective learning without data leakage, as agents respond appropriately to the realistic uncertainty within the scenarios. Furthermore, the narrower profit interquartile ranges observed for DRL agents in several cases (Fig 5) suggest greater policy robustness compared to the fixed baseline, reflecting the benefit of hourly adaptation versus adherence to a precommitted plan of the predict-then-optimize benchmark.

The differences observed between PPO and SAC are not statistically significant. Both achieve similar performance, which suggests that, in this specific case, the dominant factor is the quality of the forecasting and scenario ensemble rather than the particular actor–critic variant. We also emphasize that we evaluated three network-capacity tiers for each agent, ensuring a fair, like-for-like comparison.

## 5 Discussion

The comparison across Oracle, deterministic planning, Scenario MPC, DRL agents, and the Dummy baseline confirms our central goal: providing evidence of the practical advantages of adaptive data-driven control for PV–BESS under realistic uncertainty.

First, the DRL agents, trained on a large ensemble of probabilistic price and generation scenarios, learn closed-loop policies that react hour by hour to the realized trajectory. This contrasts with the predict-then-optimize benchmark, which commits ex ante to a single plan based on an average forecast—a future that rarely materializes exactly. The consistently higher mean profits of PPO and SAC across the three sites, together with their moderate but non-excessive cycle counts, show that DRL can exploit temporal price structure without resorting to aggressive cycling.

Second, the Scenario MPC controller plays an important role as a stochastic optimization baseline. It explicitly accounts for uncertainty through a 24-hour receding horizon and a subset of training scenarios, approximating a multistage stochastic program under realistic computational limits. Empirically, Scenario MPC outperforms the Dummy policy but remains slightly below the deterministic predict-then-optimize LP and clearly below the DRL agents. The cycle-count distributions are very similar to those of the deterministic plan, which indicates that the performance gap is not driven by “too many” or “too few” cycles, but rather by the timing of charge/discharge decisions under a short look-ahead window and a limited scenario set. In other words, under the computational budgets considered here, adding explicit stochasticity to MPC does not close the gap with DRL.

Third, the gap between the DRL agents and the perfect-foresight Oracle remains stable and reasonable across sites, which is consistent with effective learning under realistic uncertainty and provides indirect evidence against data leakage. The narrower profit interquartile ranges observed for DRL in several cases also suggest increased robustness compared to a pre-committed deterministic plan.

The differences between PPO and SAC are not statistically decisive: both algorithms achieve comparable performance when given access to the same scenario ensemble and network capacity tiers. This suggests that, in our setting, the dominant factor is the quality of the forecasting and scenario generation pipeline rather than the specific actor–critic variant. Future work could therefore focus on richer probabilistic forecasts (e.g., including extreme events) and on extending the framework to multi-asset coordination or to settings where battery degradation is explicitly modeled in the reward.

## 6 Conclusions

This study provided validation for an integrated framework for deep learning forecasting and DRL operation control for PV-BESS plants. We tested the framework using operational data from three utility-scale PV-BESS plants under real-world market conditions in Chile. Our findings bridge a gap between simulation-based research and practical deployment, offering a data-driven solution for PV-BESS management.

Our key findings, which address the research gaps identified in Sect 2, are the following:

First, we validated the proposed framework beyond the simulation; we used two years of operational data from three distinct PV-BESS plants operating under the Chilean PMGD market regime. This testing process provides evidence of the effectiveness of the model in complex and volatile real-world conditions. The better performance of the Seq2Seq forecast over traditional SARIMAX and Naive baselines was a critical prerequisite for this success. In addition, to illustrate the close connection with real-world operations, it should be noted that the company that owns these plants was familiar with the proposed methodologies and actively supported the research by granting access to its data and processes, including hosting our research team on site for several months.

Second, we show the effective integration of a Seq2Seq LSTM probabilistic forecaster with DRL control agents such as PPO and SAC. We trained these agents in a large scenario dataset, the agents learned profitable control policies that dynamically adjusted to forecasts and market prices, proving the feasibility and effectiveness of this coupled approach through empirical results (e.g. profit distributions).

Third, systematic comparison with relevant baselines, particularly the *predict-then-optimize* approach, confirmed the practical economic value of the DRL framework. The consistent better performance of DRL agents validates that adaptive policies trained in scenario distributions offer greater profitability than fixed plans. The observed contracyclical behavior further demonstrates effective learning for price arbitrage under realistic conditions.

For future research, we can mention some ideas we have. Although our DRL agents learned effective policies, incorporating Explainable AI (XAI) techniques, such as LIME or SHAP, could enhance their interpretability and help address the *black-box* nature of deep learning models [46]. Another extension would be to model battery degradation within the reward function to optimize long-term asset health alongside short-term profit; this will require a different approach to demand forecasting. Finally, the framework could be scaled to a multi-agent system to coordinate the control of a distributed fleet of PV-BESS plants, potentially enabling participation in ancillary service markets.

In conclusion, this study provides empirical evidence that a data-driven integrated AI framework with DRL can manage PV–BESS plants under real-world conditions. By delivering a reproducible framework validated on commercial-scale operational data, this work demonstrates the practical readiness of such AI-based approaches to enhance the autonomy and potential profitability of renewable energy systems.

## Supporting information

S1 Fig Price forecast results for a 4-day sample period. (TIFF)

S2 Fig Median SoC vs. mean price (normalized) for Cachiyuyo with agent PPO. (TIFF)

S3 Fig Median SoC vs. mean price (normalized) for Illapel with agent SAC. (TIFF)

S4 Fig Median SoC vs. mean price (normalized) for Illapel with agent PPO. (TIFF)

S5 Fig Median SoC vs. mean price (normalized) for Romeral with agent SAC. (TIFF)

S6 Fig Median SoC vs. mean price (normalized) for Romeral with agent PPO. (TIFF)

S7 Fig Profit distribution across scenarios — Illapel. (TIFF)

S8 Fig Profit distribution across scenarios — Romeral. (TIFF)

S9 Fig Equivalent full cycles over the horizon — Illapel. (TIFF)

S10 Fig Equivalent full cycles over the horizon — Romeral. (TIFF)

S1 AppendixSoC and price overlays.This appendix reports the state-of-charge (SoC) and price overlays for the remaining sites (Illapel and Romeral) and for both DRL agents (SAC and PPO). Each plot displays the median SoC across scenarios together with the mean nodal price, both normalized to [0,1] for visual comparison. As in the main text, the profiles show contracyclical patterns, i.e., SoC tends to be high during low-price hours and low during high-price hours. In particular, S3_FigS3 Fig and S4_FigS4 Fig report the Illapel site for agents SAC and PPO, respectively, whereas S5_FigS5 Fig and S6_FigS6 Fig show the corresponding overlays for Romeral.(PDF)

S2 Appendix Champion DRL agents. Champion DRL agents by site, capacity, and mean profit over 100 test scenarios are summarized in Table.
(PDF)

S3 Appendix Abbreviations. Table lists the abbreviations used in the paper.
(PDF)

S1 Data PV BESS Chile. (ZIP)
