# Supplementary figures and images for: Integrated forecasting and deep reinforcement learning for price-based self-scheduling of PV-BESS: Utility-scale evidence in Chile
Source: PLoS One. 2026 Jan 9;21(1):e0336753. doi: 10.1371/journal.pone.0336753 (PMC12788681; doi:10.1371/journal.pone.0336753)

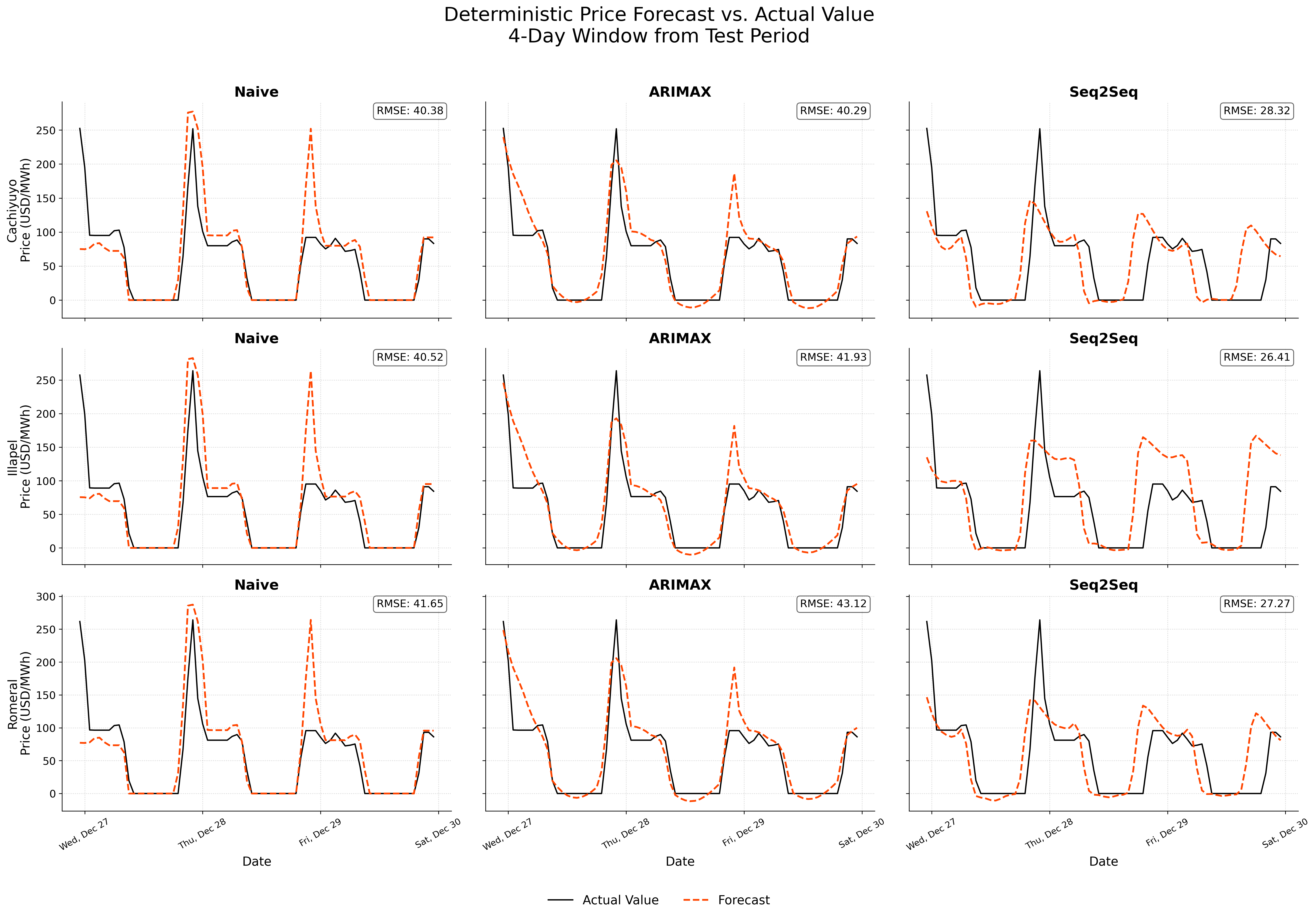

Supplement: S1 Fig — (TIFF) [file pone.0336753.s001.tif]

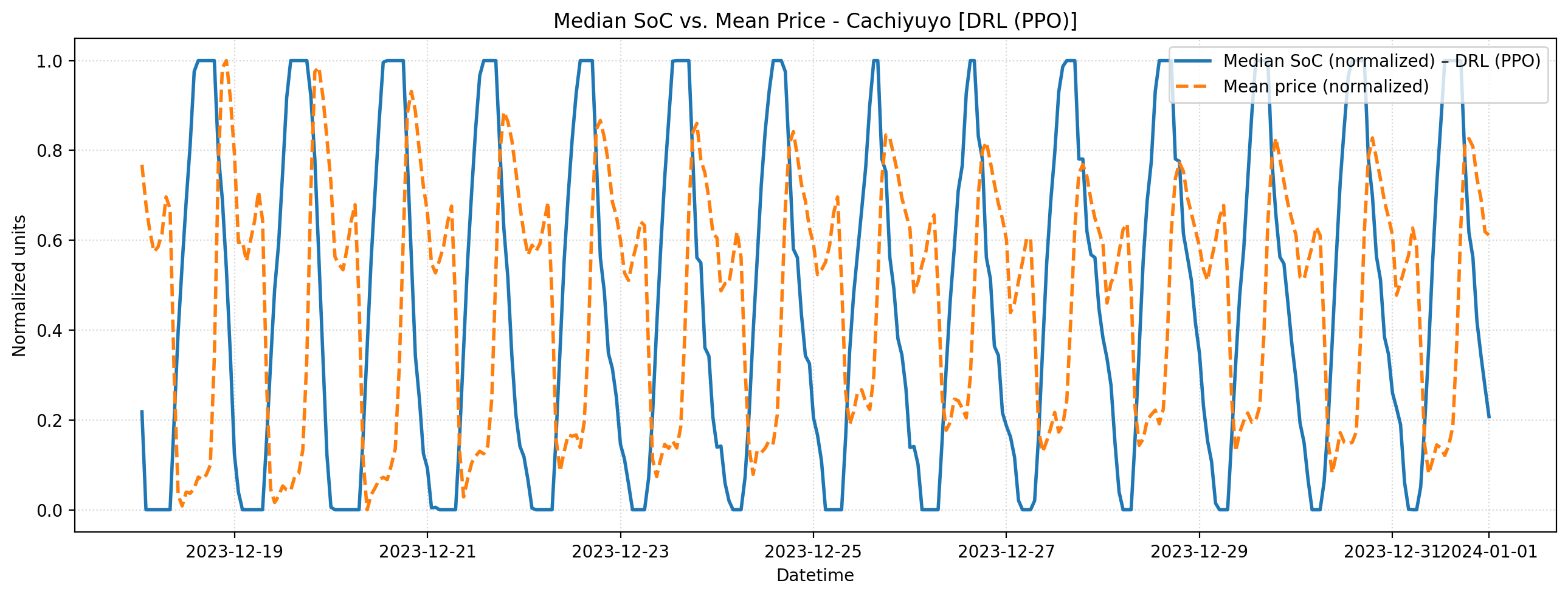

Supplement: S2 Fig — (TIFF) [file pone.0336753.s002.tif]

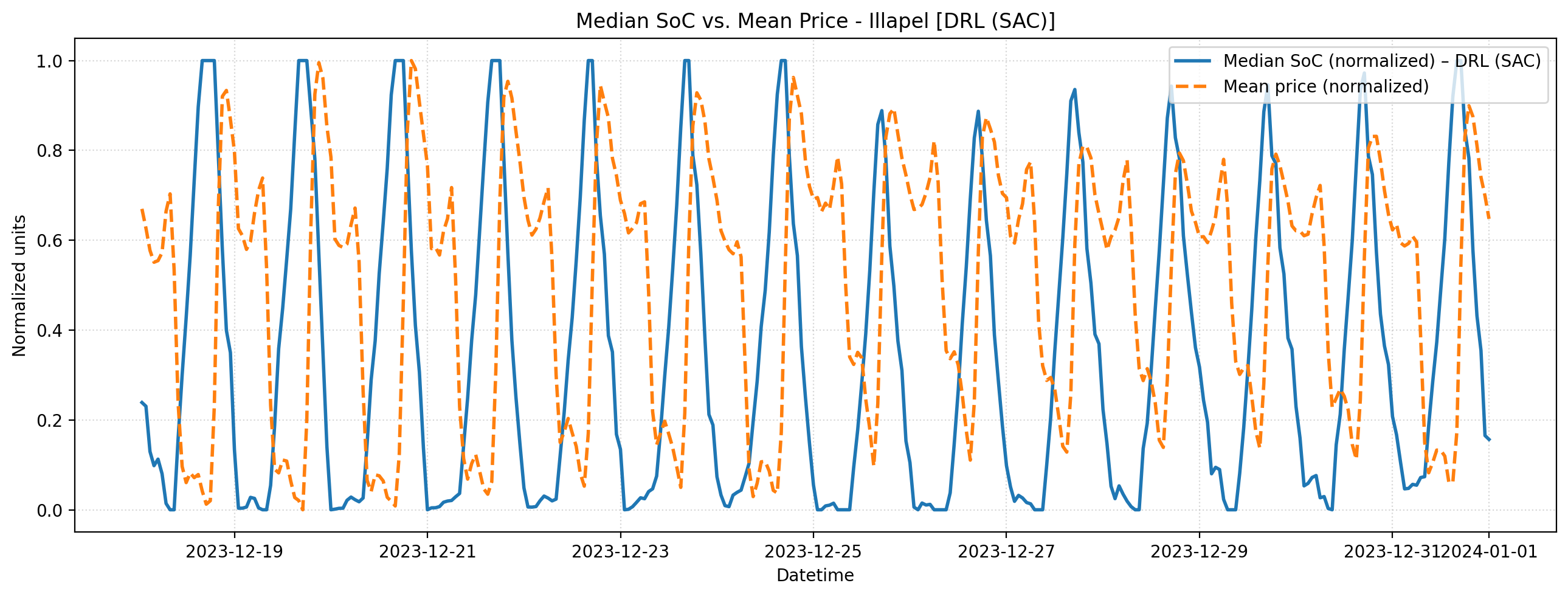

Supplement: S3 Fig — (TIFF) [file pone.0336753.s003.tif]

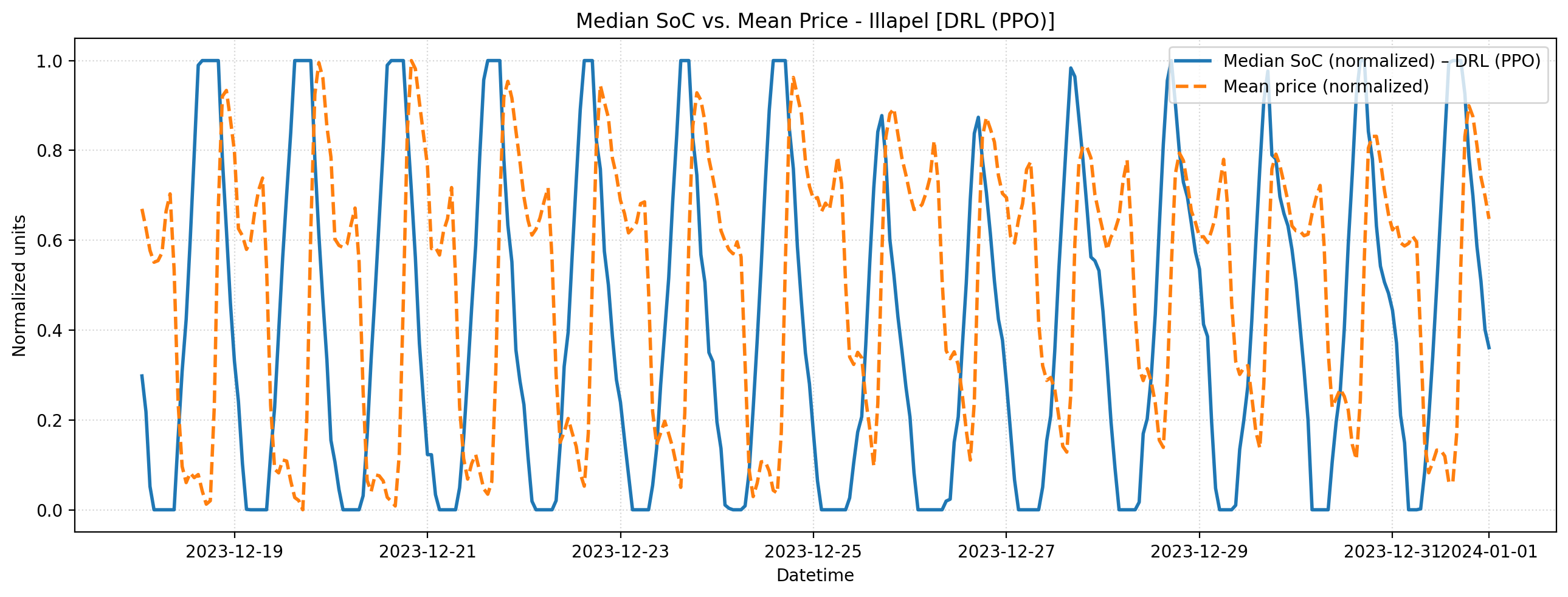

Supplement: S4 Fig — (TIFF) [file pone.0336753.s004.tif]

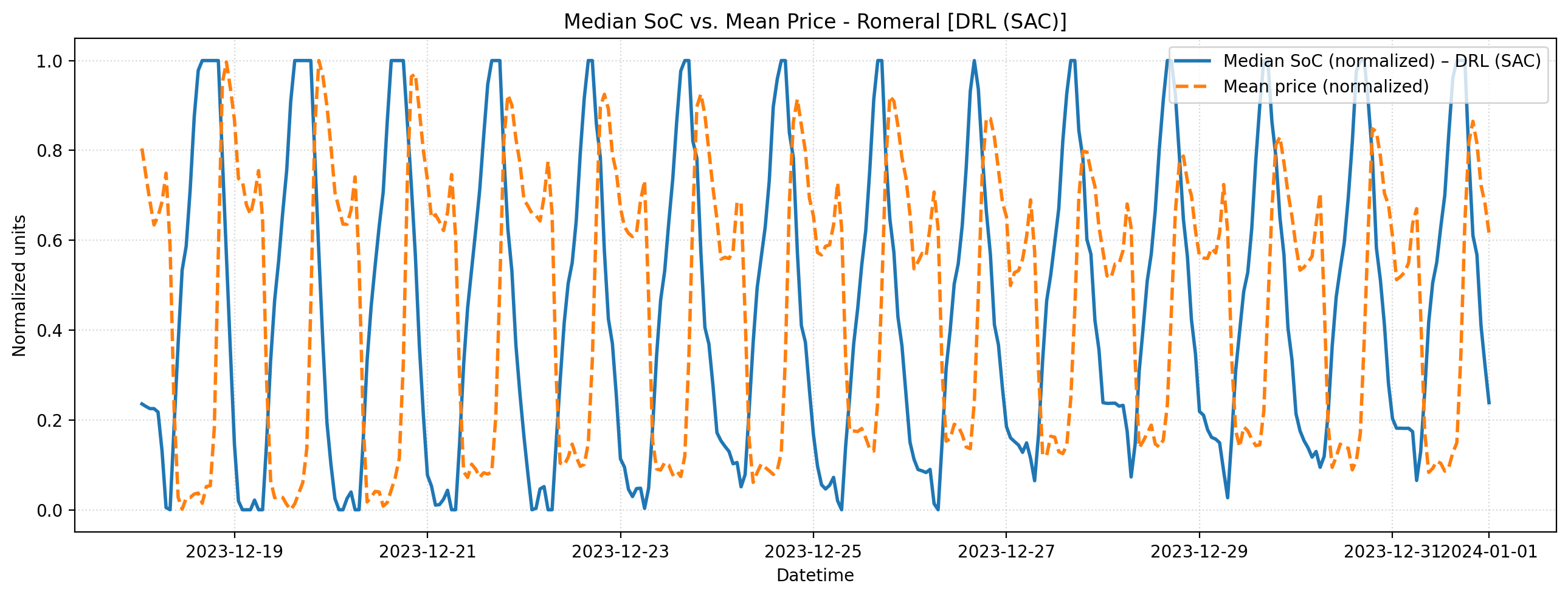

Supplement: S5 Fig — (TIFF) [file pone.0336753.s005.tif]

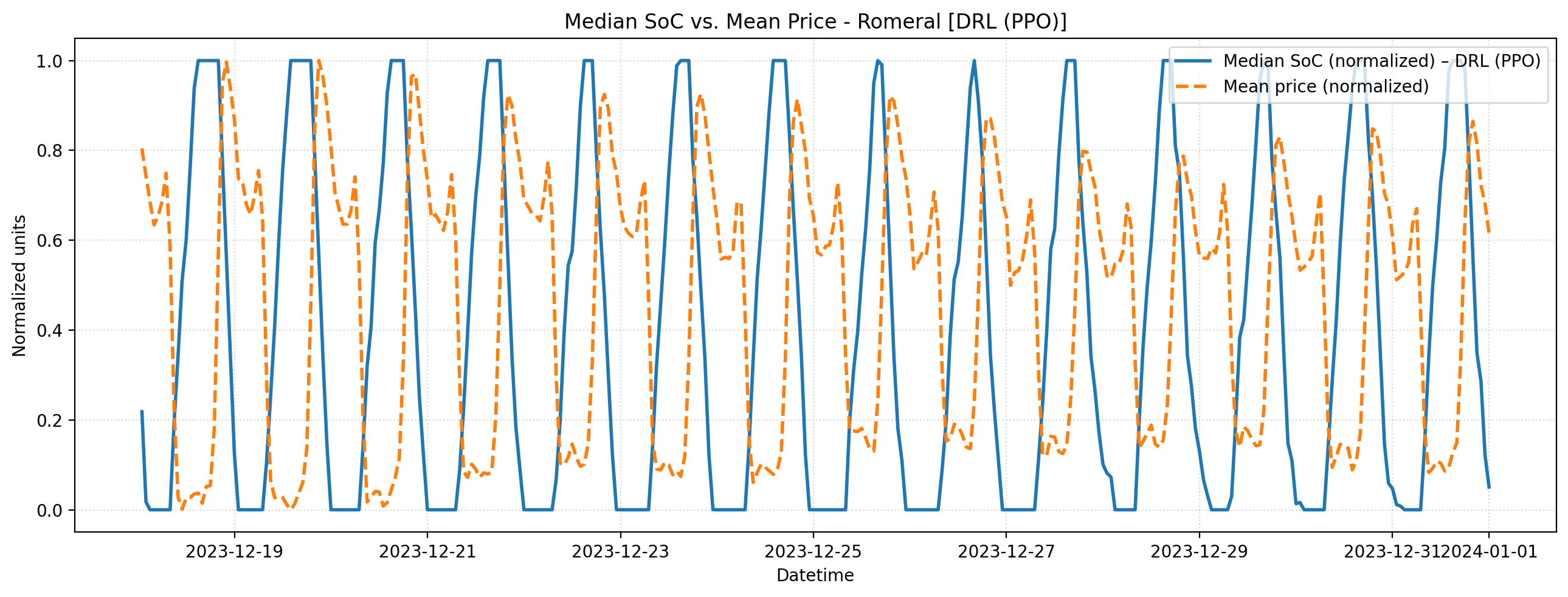

Supplement: S6 Fig — (TIFF) [file pone.0336753.s006.tif]

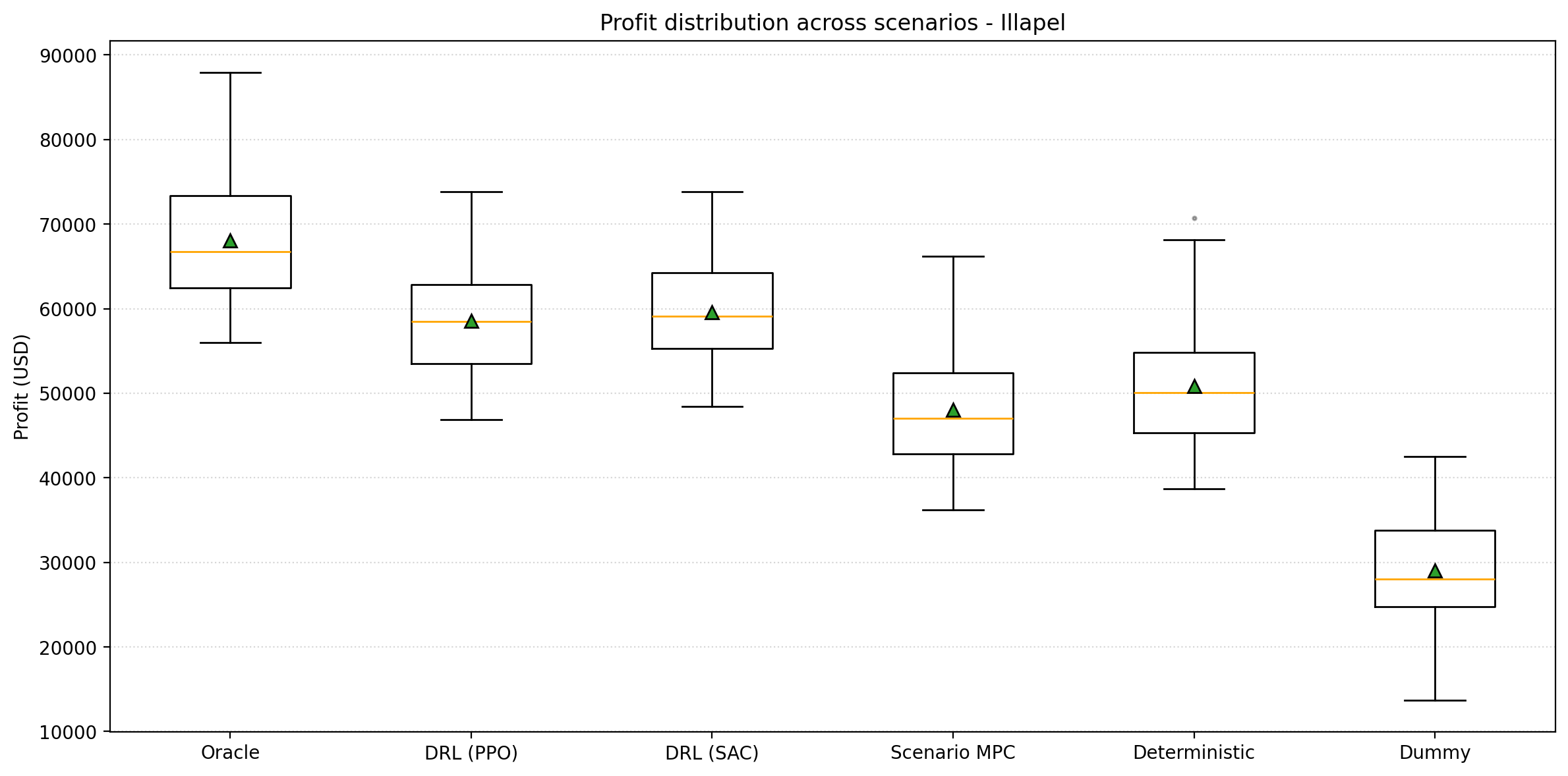

Supplement: S7 Fig — (TIFF) [file pone.0336753.s007.tif]

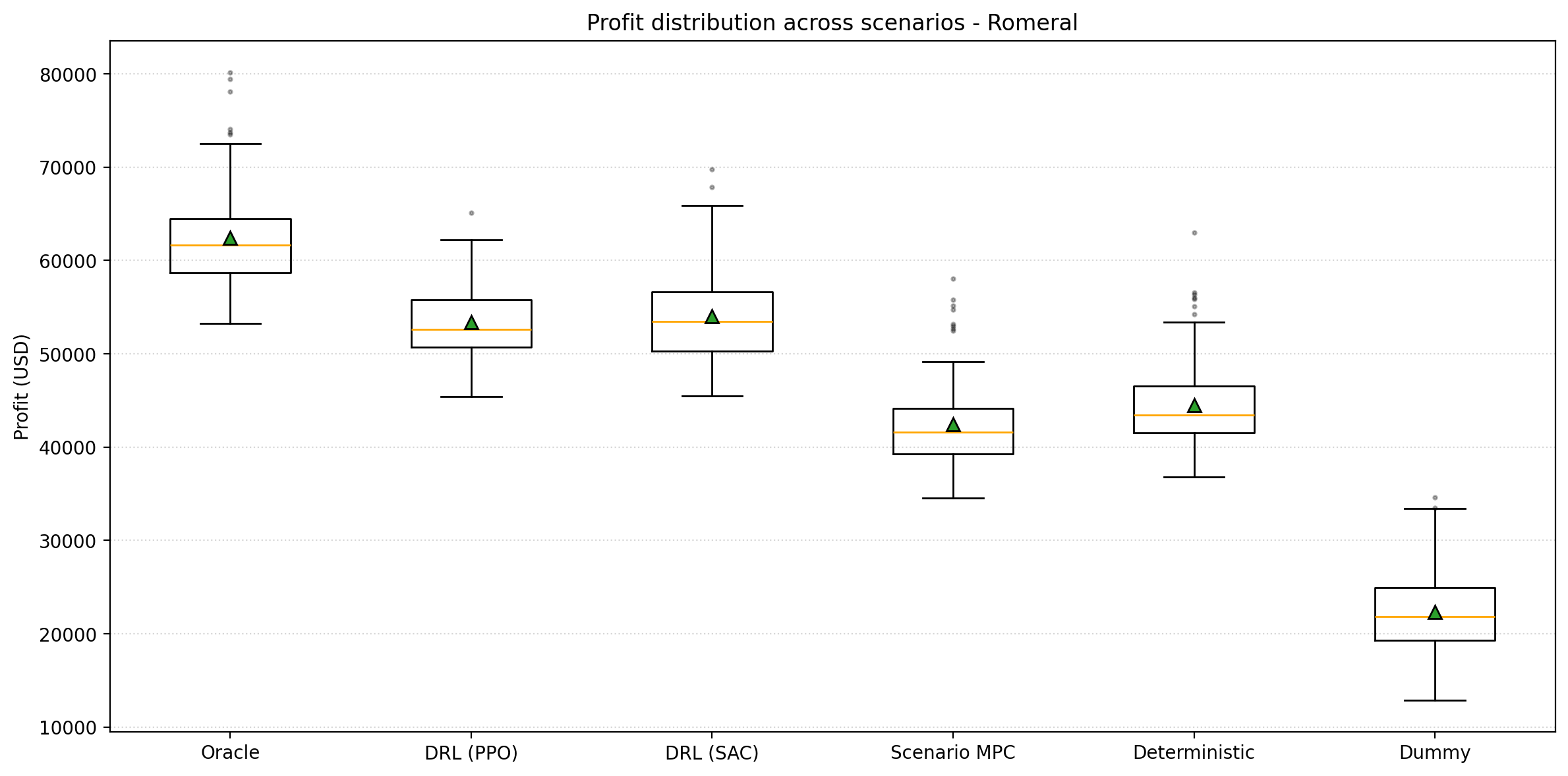

Supplement: S8 Fig — (TIFF) [file pone.0336753.s008.tif]

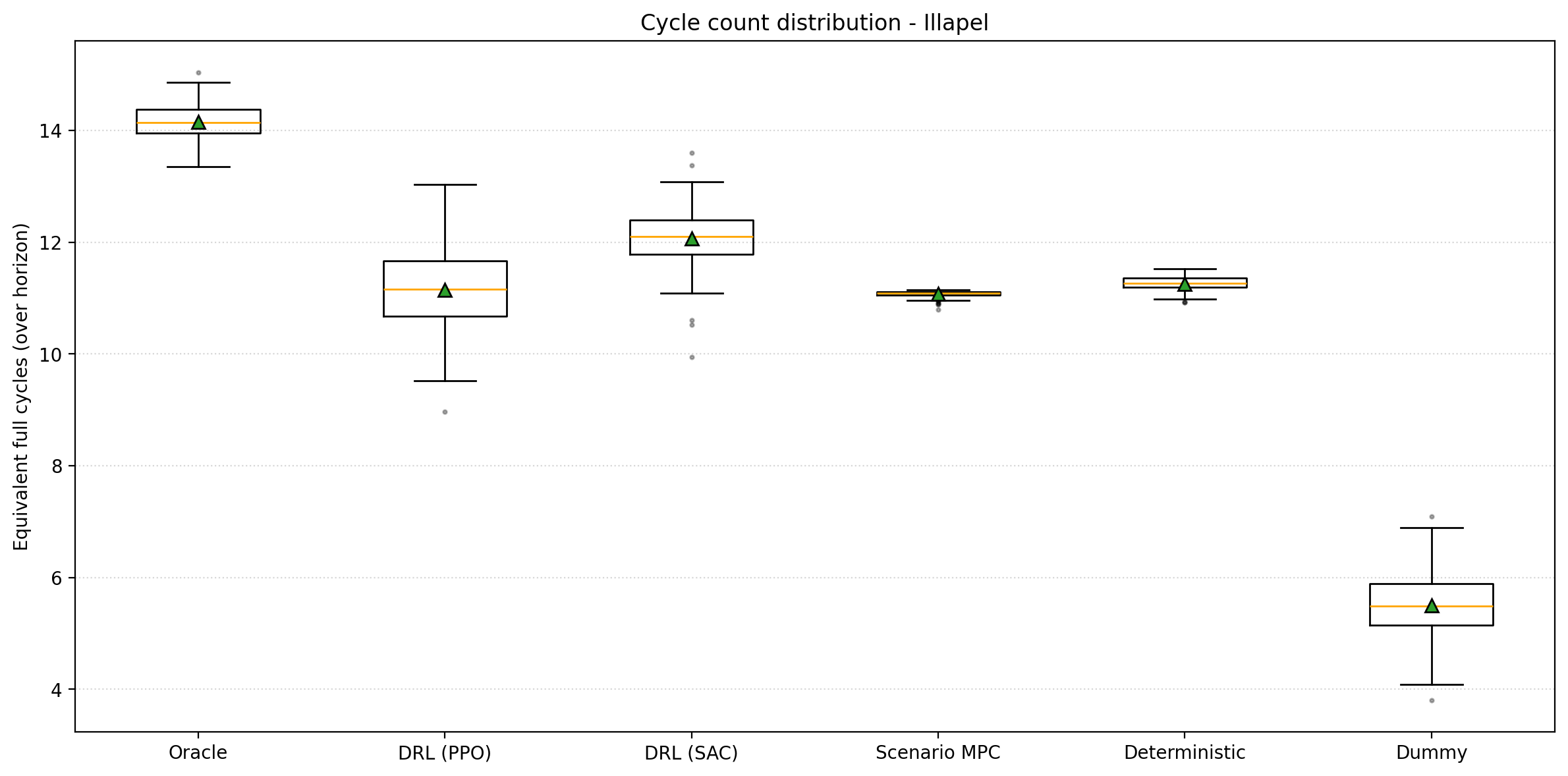

Supplement: S9 Fig — (TIFF) [file pone.0336753.s009.tif]

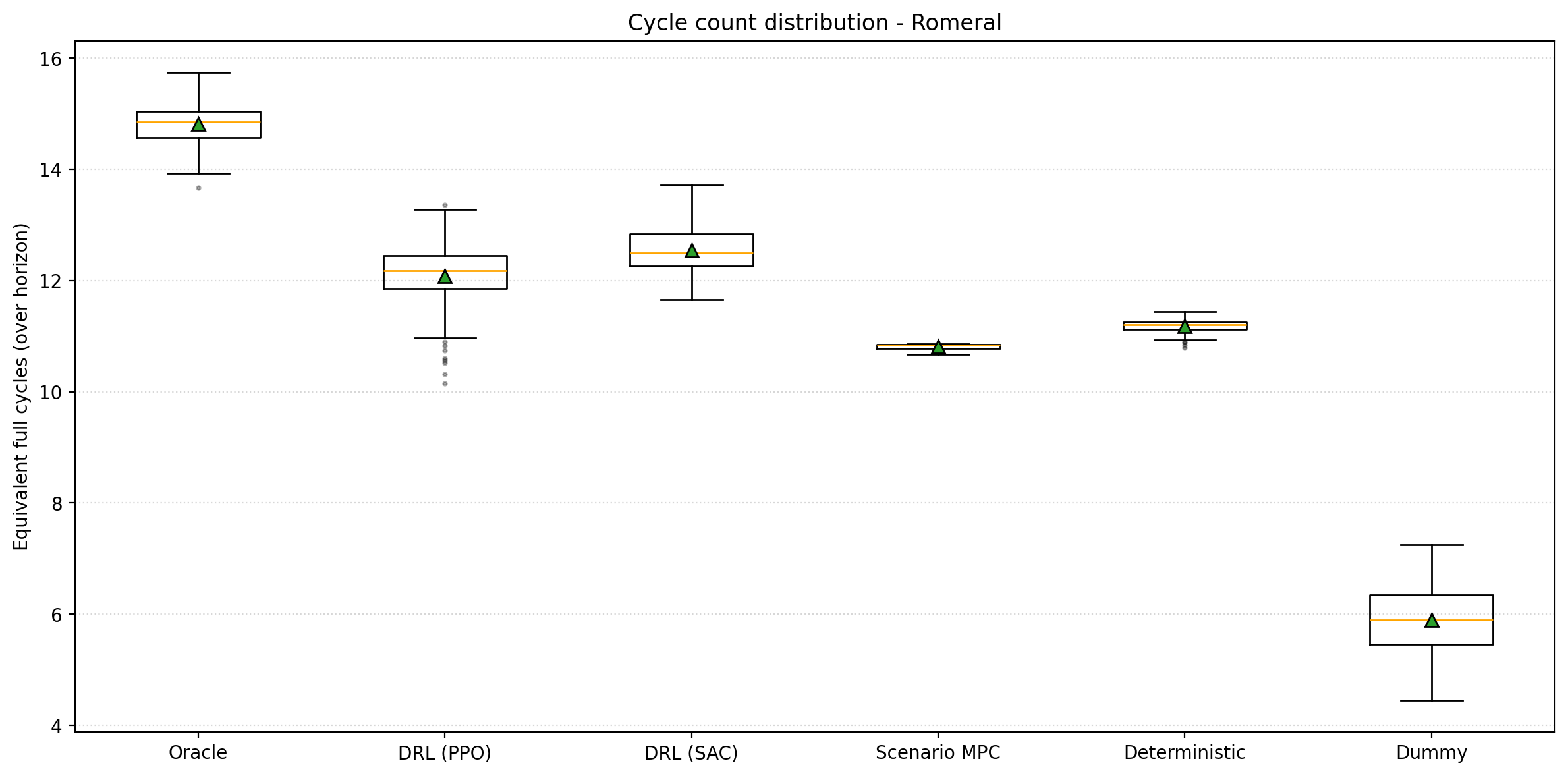

Supplement: S10 Fig — (TIFF) [file pone.0336753.s010.tif]
