# Supplementary material for: Integrated forecasting and deep reinforcement learning for price-based self-scheduling of PV-BESS: Utility-scale evidence in Chile
Source: PLoS One. 2026 Jan 9;21(1):e0336753. doi: 10.1371/journal.pone.0336753 (PMC12788681; doi:10.1371/journal.pone.0336753)
Supplement: S1 Appendix — This appendix reports the state-of-charge (SoC) and price overlays for the remaining sites (Illapel and Romeral) and for both DRL agents (SAC and PPO). Each plot displays the median SoC across scenarios together with the mean nodal price, both normalized to [0,1] for visual comparison. As in the main text, the profiles show contracyclical patterns, i.e., SoC tends to be high during low-price hours and low during high-price hours. In particular, S3_FigS3 Fig and S4_FigS4 Fig report the Illapel site for agents SAC and PPO, respectively, whereas S5_FigS5 Fig and S6_FigS6 Fig show the corresponding overlays for Romeral. (PDF) [file pone.0336753.s011.pdf]

S1 Appendix. SoC and price overlays

This appendix reports the state-of-charge (SoC) and price overlays for the remaining sites (Illapel and Romeral) and for both DRL agents (SAC and PPO). Each plot displays the median SoC across scenarios together with the mean nodal price, both normalized to $[0, 1]$ for visual comparison. As in the main text, the profiles show contracyclical patterns, i.e., SoC tends to be high during low-price hours and low during high-price hours. In particular, S3 Fig and S4 Fig report the Illapel site for agents SAC and PPO, respectively, whereas S5 Fig and S6 Fig show the corresponding overlays for Romeral.
