# Supplementary material for: Integrated forecasting and deep reinforcement learning for price-based self-scheduling of PV-BESS: Utility-scale evidence in Chile
Source: PLoS One. 2026 Jan 9;21(1):e0336753. doi: 10.1371/journal.pone.0336753 (PMC12788681; doi:10.1371/journal.pone.0336753)
Supplement: S2 Appendix — Champion DRL agents by site, capacity, and mean profit over 100 test scenarios are summarized in Table. (PDF) [file pone.0336753.s012.pdf]

S2 Appendix. Champion DRL agents

Champion DRL agents by site, capacity, and mean profit over 100 test scenarios are summarized in Table 1.

Table 1: Champion DRL agents by site, capacity, and mean profit over 100 test scenarios.

Site	Algorithm	Capacity	Mean profit (USD)
Cachiyuyo	PPO	medium	$54,428.21
Cachiyuyo	SAC	large	$52,125.77
Illapel	PPO	medium	$58,544.61
Illapel	SAC	large	$59,569.15
Romeral	PPO	medium	$53,387.37
Romeral	SAC	large	$54,033.18
