# Supplementary material for: Integrated forecasting and deep reinforcement learning for price-based self-scheduling of PV-BESS: Utility-scale evidence in Chile
Source: PLoS One. 2026 Jan 9;21(1):e0336753. doi: 10.1371/journal.pone.0336753 (PMC12788681; doi:10.1371/journal.pone.0336753)
Supplement: S3 Appendix — Table lists the abbreviations used in the paper. (PDF) [file pone.0336753.s013.pdf]

S3 Appendix. Abbreviations

Table 1 lists the abbreviations used in the paper.

Table 1: Abbreviations used in the paper.

**Abbreviation**	**Meaning**
PV	Photovoltaic
BESS	Battery energy storage system
EMS	Energy management system
RL	Reinforcement learning
DRL	Deep reinforcement learning
Seq2Seq	Sequence-to-sequence
LSTM	Long short-term memory
MPC	Model predictive control
SoC	State of charge
PPO	Proximal Policy Optimization
SAC	Soft Actor–Critic
RMSE	Root mean square error
MAE	Mean absolute error
PMGD	*Pequeños Medios de Generación Distribuida*
MDP	Markov decision process
LP	Linear programming
MLP	Multi-layer perceptron
GPU	Graphics processing unit
CSV	Comma-separated values
