# Supplementary material for: Integrated forecasting and deep reinforcement learning for price-based self-scheduling of PV-BESS: Utility-scale evidence in Chile
Source: PLoS One. 2026 Jan 9;21(1):e0336753. doi: 10.1371/journal.pone.0336753 (PMC12788681; doi:10.1371/journal.pone.0336753)
Supplement: S1 Data — (ZIP) [file pone.0336753.s014.zip › Readme.pdf]

**Title:**
Supporting data for "Integrated forecasting and deep reinforcement learning for price-based self-scheduling of PV-BESS: Utility-scale evidence in Chile"

**Description:**
This archive contains the processed datasets used in the analyses reported in the manuscript. The data correspond to three utility-scale photovoltaic plants located in Chile and include hourly PV generation, meteorological variables, and nodal electricity prices. These datasets constitute the data set required to reproduce the results, tables, and figures presented in the paper.

**1. PV Generation Data**

**File:** 01_generation_2022_2023.csv

**Description:**
Hourly photovoltaic energy generation for the studied PV plants during the 2022–2023 period.

**Typical variables include:**

- timestamp: hourly time index (local Chilean time)
- plant_id or plant_name: identifier of the PV plant
- generation_mwh: PV energy generation in MWh

These data are used as inputs to the forecasting models and to evaluate PV-BESS operational performance.

**2. Meteorological Data**

**Files:**

- 02_weather_cachiyuyo.csv
- 02_weather_illapel.csv
- 02_weather_romeral.csv

**Description:**
Hourly meteorological data associated with each PV plant location.

**Typical variables include:**

- timestamp: hourly time index (local Chilean time)
- irradiance_wm2: global horizontal irradiance ($W/m^2$)
- temperature_c: ambient temperature (°C)

These variables are used as exogenous inputs for PV generation forecasting.

**3. Nodal Electricity Price Data**

**Files:**

- 03_nodal_prices_copiapo_2022.tsv

- 03_nodal_prices_copiapo_2023.tsv
- 03_nodal_prices_diego_almagro_2022.tsv
- 03_nodal_prices_diego_almagro_2023.tsv
- 03_nodal_prices_los_vilos_2022.tsv
- 03_nodal_prices_los_vilos_2023.tsv

**Description:**
Hourly nodal marginal electricity prices obtained from the Chilean power system for the nodes associated with each PV plant.

**Typical variables include:**

- timestamp: hourly time index (local Chilean time)
- nodal_price_usd_mwh: marginal electricity price (USD/MWh)

These prices are used as the economic signal for PV-BESS self-scheduling and revenue calculation.

**4. Notes on Pre-processing**

- All datasets are provided at an hourly resolution.
- Time zones are consistent across generation, weather, and price data.
- The data correspond to the processed versions used directly in the analyses reported in the manuscript.
- No personal, confidential, or commercially sensitive information is included.

**5. Reproducibility**

The datasets contained in this archive are sufficient to:

- Reproduce the numerical results reported in tables.
- Reconstruct the data underlying the figures.
- Replicate the study findings described in the manuscript.
